# Zika virus modulates mitochondrial dynamics, mitophagy, and mitochondria-derived vesicles to facilitate viral replication in trophoblast cells

**DOI:** 10.3389/fimmu.2023.1203645

**Published:** 2023-09-14

**Authors:** Jae Kyung Lee, Ok Sarah Shin

**Affiliations:** BK21 Graduate Program, Department of Biomedical Sciences, College of Medicine, Korea University Guro Hospital, Seoul, Republic of Korea

**Keywords:** zika virus (ZIKV), nonstructural protein 4A (NS4A), mitochondrial quality control, mitophagy, mitochondria-derived vesicles (MDVs)

## Abstract

Zika virus (ZIKV) remains a global public health threat with the potential risk of a future outbreak. Since viral infections are known to exploit mitochondria-mediated cellular processes, we investigated the effects of ZIKV infection in trophoblast cells in terms of the different mitochondrial quality control pathways that govern mitochondrial integrity and function. Here we demonstrate that ZIKV (PRVABC59) infection of JEG-3 trophoblast cells manipulates mitochondrial dynamics, mitophagy, and formation of mitochondria-derived vesicles (MDVs). Specifically, ZIKV nonstructural protein 4A (NS4A) translocates to the mitochondria, triggers mitochondrial fission and mitophagy, and suppresses mitochondrial associated antiviral protein (MAVS)-mediated type I interferon (IFN) response. Furthermore, proteomics profiling of small extracellular vesicles (sEVs) revealed an enrichment of mitochondrial proteins in sEVs secreted by ZIKV-infected JEG-3 cells, suggesting that MDV formation may also be another mitochondrial quality control mechanism manipulated during placental ZIKV infection. Altogether, our findings highlight the different mitochondrial quality control mechanisms manipulated by ZIKV during infection of placental cells as host immune evasion mechanisms utilized by ZIKV at the placenta to suppress the host antiviral response and facilitate viral infection.

## 1 Introduction

Association between Zika virus (ZIKV) infection, pregnancy complications, and congenital neurodevelopmental disorders like microcephaly declared ZIKV a global public health threat (1). Although ZIKV is predominantly transmitted to humans by mosquitoes, accumulating evidence indicates that ZIKV is also capable of transmission via blood transfusions, sexual, or congenital routes (2). The current lack of ZIKV-specific therapies or vaccines further emphasizes the importance of uncovering molecular mechanisms underlying ZIKV infections, especially during vertical transmission.

Many viruses hijack cellular organelles like the mitochondria to facilitate their life cycle. ZIKV has been shown to manipulate the host cell machinery by remodeling cellular organelles, such as the endoplasmic reticulum and mitochondria for viral replication and immune evasion (3). Mitochondria, in particular, provide a signaling platform for activation of inflammatory and innate immune responses, and upregulation of mitochondrial fusion during retinoic acid inducible gene-I (RIG-I)-like receptor (RLR) signaling enhances the transmission of antiviral signals (4). Therefore, the quality and quantity of mitochondria are regulated by various quality control mechanisms that maintain the morphology, function, integrity, and number of mitochondria. Mitochondrial quality control mechanisms include changes in mitochondrial dynamics via fusion and fission, selective removal of mitochondria via lysosomal degradation (mitophagy), and formation of mitochondria-derived vesicles (MDVs) that can contain various mitochondrial components for degradation or extracellular release (5, 6). How ZIKV affects the mitochondrial quality control system of placental cells, however, is a question that remains to be answered.

The multifaceted roles of flavivirus NS4A during infection of host cells have previously been reported. Notably, ZIKV NS4A was identified as a major suppressor of RLR-mediated type I IFN production by disrupting the interaction between MAVS and melanoma differentiation associated protein 5 (MDA5) or RIG-I (7). In human neural stem cells (NSCs), upregulation of mitochondrial fragmentation by ZIKV NS4A contributed to ZIKV-induced neuronal cell death, and cooperation of ZIKV NS4A and NS4B was also observed during impairment of neurogenesis via inhibition of Akt-mTOR signaling, which in turn induced autophagy and enhanced viral replication (8, 9). Also, ZIKV NS4A has been reported to directly interact with ANKLE2, which is a protein linked to human microcephaly, and disrupt neural stem cell division and brain development (10). Although these findings demonstrate the importance of ZIKV NS4A in host immune evasion and viral pathogenesis, it is still unclear whether ZIKV NS4A triggers changes in mitochondrial morphology or function.

The placenta releases extracellular vesicles (EVs) of various sizes into the maternal circulation during pregnancy, and changes in levels of EVs have been associated with pregnancy-associated complications, including preterm birth, miscarriage, and preeclampsia (11, 12). Small EVs (sEVs), which are 50-150 nm in diameter and carry a variety of cargo like nucleic acids, proteins, and lipids, can modulate viral pathogenesis by carrying viral or host cellular factors that can affect viral replication and transmission (13, 14). Furthermore, placenta-derived sEVs are key modulators of maternal immune tolerance of the fetus and defense against pathogens during pregnancy, and contribute to immune cell activation, differentiation, and maturation (15). For example, ZIKV infection of a primate trophoblast cell model, which was representative of the human placenta during the early first trimester, revealed alterations in the protein and microRNA (miRNA) content of EVs secreted upon ZIKV inoculation (16). These findings demonstrate the importance of placenta-derived sEVs as important communicators at the maternal-fetal interface and their potential as biomarkers of pregnancy complications, including ZIKV-induced birth defects.

In this study, we investigated how ZIKV manipulated mitochondrial quality control mechanisms during infection of JEG-3 trophoblast cells. Furthermore, compositional analyses of sEVs secreted by ZIKV-infected JEG-3 in terms of the miRNA and protein components revealed the enrichment of mitochondrial proteins in ZIKV-infected sEVs. We performed small RNA-sequencing and proteomics analysis of sEVs secreted by ZIKV-infected JEG-3, and identified sEV-specific mitochondrial components, indicating that the mitochondria provide cargo to be encapsulated within MDVs that are then secreted as sEVs during infection. Our findings demonstrate that ZIKV mediates changes in mitochondrial dynamics, mitophagy, and MDVs to evade the host innate immune response and facilitate viral infection.

## 2 Materials and methods

### 2.1 Cells, viruses, and drugs

JEG-3 choriocarcinoma cells were purchased from Korean Cell Line Bank (South Korea) and HEK293T and Vero cells were purchased from American Type Culture Collection (ATCC). HeLa cells that stably express mitochondrial-targeted Keima (HeLa-mtKeima) cells were provided by Prof. Jeanho Yun (College of Medicine, Dong-A University, South Korea). JEG-3, Vero, HeLa, and HEK293T cells were cultured in Dulbecco’s modified Eagle’s medium supplemented with 10% fetal bovine serum (FBS) (Corning Mediatech) and 1% non-essential amino acids (Sigma). Human neural progenitor cells (hNPCs) were generated as previously described (17–19).

PRVABC59 (PRV, Asian lineage) and MR766 (African lineage) of ZIKV were purchased from ATCC, while Zika/Brazil/16321 strain (Asian lineage) was kindly provided by Korean Centers for Disease Control and Prevention. ZIKV was propagated in Vero cells as previously described (20). ZIKV infection was carried out at multiplicity of infection (MOI) of 10 for subsequent experiments. For the measurement of tissue culture infective dose (TCID_50_), supernatants from ZIKV-infected JEG-3 or HeLa cells were collected at 48 hours post infection (hpi). TCID_50_ was determined using the Spearman-Kärber method and expressed as TCID_50_/mL.

Carbonyl cyanide 3-chlorophenylhydrazone (CCCP), mitoTEMPO, Mdivi-1, Rapamycin, and 3-MA were purchased from Sigma (St. Louis, MO, USA) and staurosporine (STS) was purchased from Tocris (Bristol, UK). Mitochondria/Cytosol Fractionation kit was purchased from Abcam (Cambridge, UK).

### 2.2 Transfection of plasmids and siRNAs

A plasmid encoding mitochondrial matrix-targeted DsRED (mtDsRED) was provided by Dr. Woong Sun (Korea University School of Medicine, South Korea) and flag-tagged ZIKV NS4A and ZIKV NS4B plasmids were provided by Dr. Vaithi Arumugaswami (Addgene plasmid # 79636 and 79640). A plasmid encoding mtKeima was provided by Prof. Jeanho Yun (College of Medicine, Dong-A University, South Korea) and described in previous studies (21). Transfection with negative control, PINK1-, BNIP3-, or NIX-specific siRNAs (Bioneer, South Korea) was carried out using Lipofectamine RNAiMAX transfection reagent (Invitrogen) according to manufacturer’s instructions. hNPCs seeded in 96-well plate were transfected with varying concentrations of ZIKV NS4A-encoding plasmid using Neon transfection system (Invitrogen).

### 2.3 Confocal microscopy

MitoTracker Green (500 nM) and LysoTracker Red (75 nM), purchased from Thermo Fisher Scientific, were used for fluorescent staining of mitochondria and lysosomes, respectively. MitoSOX (250 nM) was used to visualize mitochondrial ROS. ZIKV was stained with anti-dsRNA Antibody, clone J2 (Merck; 1:200 dilution), while FLAG-tagged ZIKV NS4A was detected with anti-DYKDDDDK (FLAG) tag antibody (FUJIFILM Wako Chemical Corp.; 1:500 dilution), followed by anti-mouse FITC conjugated antibody (Cell signaling, 1:250 dilution). Coverslips were mounted on glass slides after staining with 4,6-diamidino-2-phenylindole (DAPI) (Sigma) and examined using a confocal microscope (LSM900; Carl Zeiss, Oberkochen, Germany).

### 2.4 Immunoblot analysis

Cell lysates were resolved by SDS-PAGE on 8-15% acrylamide gels and transferred onto polyvinylidene difluoride membranes. Membranes were incubated overnight with the following primary antibodies: ZIKV NS1 (Genentech, CA, USA); MFN1, OPA1, phospho-Drp1 (Ser616), Drp1, LC3, p62, cleaved/total Caspase 3, cleaved/total PARP, PINK1, Parkin, BNIP3, NIX (Cell Signaling Technology, MA, USA); GFP, IRF3, Tom20, G3BP1, LAMP1 (Santa Cruz biotechnology, San Diego, CA, USA)); TOM70, Snx6, Snx9 (Proteintech, IL, USA); FLAG tag (Fujifilm Wako Pure Chemical Corporation, Osaka, Japan), myc, β-actin (Abcepta, CA, USA); MAVS/VISA (Bethyl laboratories, MA, USA). After incubation with secondary antibodies, membranes were imaged using Fusion Solo Imaging System (Vilber Lourmat Sté, Collégien, France), and band intensities were quantified with ImageJ (National Institutes of Health, Bethesda, MD).

### 2.5 Co-immunoprecipitation

HeLa cells seeded in 6-well plates were transfected with the indicated plasmids and incubated for 24 h. Then, cells were scraped and resuspended in Pierce IP Lysis Buffer (Thermo Fisher Scientific, Waltham, MA, USA) supplemented with a protease inhibitor cocktail (Sigma). After removing cell debris via centrifugation (16,000 x g, 4°C, 15 min), primary antibodies were incubated with 50 μl protein A magnetic beads (SureBeads, Bio-Rad, Hercules, CA, USA) with rotation. Beads were washed extensively with PBS supplemented with 0.1% Tween20 and incubated with 300 μg proteins at 4°C with rotation. Proteins were analyzed by immunoblot analysis. Protein samples were probed with the following primary antibodies: FLAG tag (1:2000; Fujifilm Wako), GFP tag (1:500; Santa Cruz)

### 2.6 Quantitative real-time RT polymerase chain reaction (qRT-PCR)

Direct-zol RNA mini kit (Zymo Research, Irvine, CA, USA) was used for total RNA extraction, followed by cDNA synthesis using ImProm-II Reverse Transcription System (Promega, Madison, WI, USA) according to manufacturer’s instructions. mRNA expression levels were quantified using Power SYBR Green Master Mix (Invitrogen) and QuantStudio 6 Flex real-time PCR system (Thermo Fisher Scientific) under the following conditions: 95°C for 10 min, followed by 40 cycles of 95°C for 30 s and 60°C for 1 min. The results are presented as fold change from matched mock-infected controls calculated using the 2^−ΔΔCT^ method normalized to GAPDH. Primer sequences are provided in **Supplementary Table 1**. For mitochondrial DNA (mtDNA) quantification, total or cytosolic genomic DNA was extracted from cells using phenol-chloroform and ethanol method and subjected to PCR (22). Relative level of mtDNA was determined based on the 2^−ΔΔCT^ method normalized against 18s rRNA.

### 2.7 Mitochondrial metabolic flux

JEG-3 cells were seeded at 12,000 cells per well using reduced serum-supplemented DMEM medium. For transfection, empty vector (EV) or ZIKV NS4A-encoding plasmids were used for transfection at 100 ng per plasmid using Lipofectamine 2000. Cells were incubated in a CO_2_-free incubator at 37 °C for 1 h to allow for temperature and pH equilibration before being loaded into the XFp analyzer. Under these basal conditions, 1 μM oligomycin, 2 µM fluoro-carbonyl-cyanide phenylhydrazone (FCCP) or 1 μM rotenone and 1 μM antimycin A (Rot/Ant) were injected at the indicated time points. Mitochondrial Stress test kit was applied to determine oxygen consumption rate (OCR) using Seahorse XF HS Mini analyzer (Agilent Technologies, Santa Clara, CA, USA).

### 2.8 Mitochondrial membrane potential (MMP) measurement

Cells seeded in 96-well black plates were transfected with ZIKV NS4A-encoding plasmid at varying concentrations. After 24 h, cells were incubated with fluorescent dye tetramethylrhodamine methyl ester (TMRM) (Thermo Fisher Scientific) (20 nM, 30 min) at 37°C and washed with Dulbecco’s phosphate-buffered saline (DPBS). TMRM fluorescence was measured using Varioskan microplate reader (Thermo Fisher Scientific).

### 2.9 Mitochondrial reactive oxygen species (mtROS) measurement

JEG-3 cells seeded in 96-well black plates were treated with mitochondrial ROS scavenger mitoTEMPO (1 μM) for 16 h, prior to transfection with ZIKV NS4A-encoding plasmid. At 24 h post-transfection, cells were treated with CCCP, which is a commonly used mitophagy inducer that causes mitochondrial depolarization, and then incubated with mitoSOX for 45 min. mitoSOX fluorescence was analyzed with Varioskan microplate reader.

### 2.10 Luciferase reporter assay

Luciferase reporter assay was performed as described previously (20). JEG-3 or HEK293T cells seeded in 96-well plates were transfected with IFN-β and ISRE reporter plasmids expressing firefly luciferase in combination with plasmids encoding empty vector, ZIKV NS4A, ZIKV NS4B, MDA5, RIG-I-CARD, or MAVS using Lipofectamine 2000 (Invitrogen). At 24 h post-transfection, cells were harvested and cell lysates were assessed for luciferase activities using Dual-Glo Luciferase Assay Kit (Promega).

### 2.11 sEV isolation and characterization

When JEG-3 cells reached 70-80% confluence in 100 mm dishes, they were washed with PBS prior to infection and incubated with serum-free DMEM following removal of the virus. Conditioned medium of mock- or ZIKV-infected cells were collected at 48 hours post infection (hpi) and used for isolation and purification of sEVs using Exo-Spin kit (Cell Guidance Systems) in accordance with the manufacturer’s instructions. sEVs were characterized according to the guidelines provided by the Minimal Information for Studies of Extracellular Vesicles (14). NanoSight NS300 (Malvern Panalytical) was used for nanoparticle tracking analysis of mean sEV concentration and diameter.

Immunoblot analysis of sEV-specific markers was performed using sEVs prepared in exosome resuspension buffer (Thermo) containing a protease inhibitor cocktail (Roche Applied Science). Lysates were subjected to SDS-PAGE and transferred to polyvinylidene difluoride membranes, which were incubated with primary antibodies against calnexin (Cell Signaling Technology), ALIX (System Biosciences), TSG101 (Proteintech), CD9 (Proteintech), and CD63 (Santa Cruz) overnight at 4°C, followed by incubation with horseradish peroxidase−conjugated anti-rabbit or anti-mouse IgG secondary antibodies for 1 hour at room temperature. Membranes were then incubated with ECL solution (Thermo Fisher Scientific) for visualization.

Transmission electron microscopy was used to confirm sEV size and morphology. sEVs isolated from mock- or ZIKV-infected JEG-3 cells were mounted on formvar/carbon-coated nickel grids and stained with 5% uranyl acetate for negative staining of the vesicles. Samples were washed with PBS and dried prior to imaging with Tecnai G2 Spirit TWIN TEM (FEI, Netherlands) at accelerating voltage of 120 kV.

### 2.12 Small RNA sequencing and miRNA-mRNA target prediction analysis

Total RNA was extracted using Trizol reagent (Invitrogen) according to the manufacturer’ instructions. RNA quality was assessed by Agilent 2100 bioanalyzer using the RNA 6000 PicoChip (Agilent Technologies), and RNA quantification was performed using a NanoDrop 2000 Spectrophotometer (ThermoFisher Scientific). Library was constructed with 1 μg of the total RNA from each sample using NEBNext Multiplex Small RNA Library Prep kit (New England BioLabs) according to the manufacturer’s instructions. NextSeq500 system was utilized to generate high-throughput sequences (single-end reads; 75 cycles) (Illumina). Mature miRNA sequence was used as a reference for mapping sequence reads with bowtie 2 software. miRNA expression levels for each sample were determined using the read counts, and quantile normalization method was used to compare different samples. For miRNA-mRNA target prediction and analysis, miRWalk 2.0, miRTarBase, TargetScan, and miRDB were utilized. Small RNA-sequencing data are accessible via GEO under accession number GSE228310.

### 2.13 Liquid chromatography with tandem mass spectrometry (LC–MS/MS) analysis-based proteomics profiling and bioinformatics analysis

sEV protein samples were suspended in lysis buffer supplemented with a protease inhibitor cocktail and sonicated for 20 min. BCA protein assay kit (Pierce) was used to quantify protein concentration. Samples were prepared using Filter-Aided Sample Preparation (FASP) digestion method. The digested peptides were desalted using C18 spin columns (Harvard Apparatus), and the peptides were eluted with 80% acetonitrile in 0.1% formic acid in water. Peptides were analyzed with Q-Exactive Orbitrap hybrid mass spectrometer (Thermo) and Ultimate 3000 (U)HPLC systems (Thermo) to obtain proteomics data. Samples were prepared in duplicates and total of 4 runs were performed. Proteome Discoverer software (ver. 2.5) was used to identify and quantify proteins present in sEVs and Uniprot was used to download the *homo sapiens* database and predict the subcellular localization of the identified proteins. Fold change was calculated based on protein abundance. Gene Ontology terms associated with the genes encoding the identified proteins were analyzed to identify and group molecular functions of differentially regulated sEV proteins (fold change > 2, p-value < 0.05).

### 2.14 Statistical analysis

An unpaired two-tailed Student’s t-test or a Mann−Whitney test was performed using GraphPad Prism 9.0 (GraphPad Software, San Diego, CA, USA).

## 3 Results

### 3.1 ZIKV NS4A modulates mitochondrial dynamics and respiration profile, and induces mtROS production

**Figure 1:**
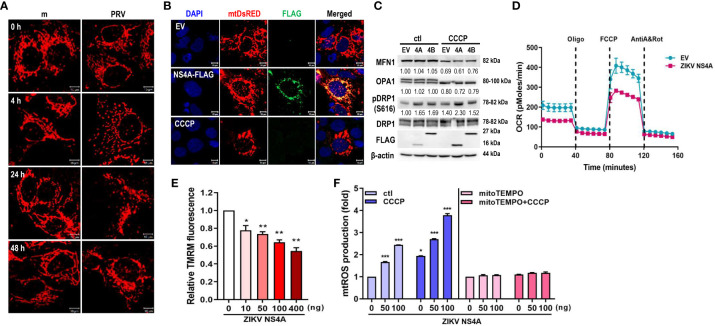
ZIKV NS4A disrupts mitochondrial dynamics and respiration profile, and induces mtROS production. **(A)** JEG-3 cells were transfected with mtDsRED-encoding plasmid. Next day, cells were mock-infected or infected with PRVABC59 (PRV) (MOI 10) and fixed at the indicated time points to visualize mitochondrial morphology using confocal microscopy. Scale bar = 10 μm. **(B)** ZIKV NS4A-induced mitochondrial fragmentation was visualized using JEG-3 cells transfected with mtDsRED and empty vector (EV) or FLAG-tagged ZIKV NS4A plasmids. Scale bar = 10 μm. CCCP-treated cells were included as a control for mitochondrial fragmentation. **(C)** Western blot analysis of proteins regulating mitochondrial dynamics in JEG-3 cells overexpressing EV, ZIKV NS4A or NS4B. Band intensities were quantified using ImageJ software and normalized against β-actin. **(D)** Real-time measurement of mitochondrial stress assay for EV or ZIKV NS4A-overexpressing cells was carried out to determine ZIKV NS4A-induced changes in oxygen consumption rate (OCR) following treatment with oligomycin (1.5 μM), FCCP (2μM) or rotenone + Antimycin A (0.5μM each) **(E)** Tetramethylrhodamine methyl ester (TMRM) assay of JEG-3 cells overexpressing increasing concentrations of ZIKV NS4A-encoding plasmid. NS4A-induced mitochondrial membrane depolarization was determined using fluorescence microplate reader. *p<0.05, **p<0.01, when compared to the control. **(F)** JEG-3 cells were transfected with different amount of ZIKV NS4A-encoding plasmid. Cells were treated with DMSO control (ctl), CCCP, or mito-TEMPO, an antioxidant targeted to mitochondria. Mitochondrial ROS (mtROS) was measured by MitoSox Red staining and mtROS levels were quantitatively determined using fluorescence analysis software (mean ± SD; n = 3).*p<0.05, ***p<0.001, when compared to the control.

To investigate the impact of ZIKV infection on host mitochondrial dynamics, we first examined the effect of ZIKV infection on JEG-3 mitochondrial morphology. Confocal imaging of mock-infected JEG-3 cells revealed networks of elongated mitochondrial filaments while PRV-infected cells contained mitochondria that exhibited round morphology and shortened filaments starting at 4 hpi, similar to CCCP-treated cells (**Figure 1A**). Furthermore, PRV-induced fragmentation of mitochondrial filaments was reversed or exacerbated upon knockdown of fission factor, Drp1, or fusion factor, MFN2, respectively, and inhibition of Drp1-mediated fission via mdivi-1 treatment also relieved PRV-induced fission (**Supplementary Figure 1A**).

To study the potential role of ZIKV NS4A during PRV-mediated changes of mitochondrial morphology and dynamics, we co-transfected JEG-3 cells with mtDsRED and ZIKV NS4A-encoding plasmids. Analysis of fluorescence microscopy revealed co-localization of ZIKV NS4A and mtDsRED, indicating mitochondrial localization of ZIKV NS4A. In addition, CCCP treatment and ZIKV NS4A overexpression resulted in mitochondrial fragmentation when compared to empty vector-transfected cells (**Figure 1B**). ZIKV NS4A also modulated expression levels of proteins involved in regulating mitochondrial dynamics, in particular, phosphorylation of Drp1 at Ser616 site was increased, which is a posttranslational modification necessary for Drp1 recruitment to the mitochondria and subsequent activation of fission (23) (**Figure 1C**). Differences in mitochondrial oxygen consumption were also observed in JEG-3 cells following transfection with empty vector- or ZIKV NS4A-encoding plasmids (**Figure 1D**). Basal and maximal respiration were both reduced in ZIKV NS4A-transfected cells. ZIKV NS4A overexpression was also associated with depolarization of mitochondrial membrane, as shown by the decrease in mitochondrial membrane potential (MMP) (**Figure 1E**). ROS production can also result from MMP depolarization, and mitochondria-driven ROS production can trigger cellular stress and activate host innate immunity (24). ZIKV NS4A induced mitochondrial ROS production, which was abrogated upon treatment with mitochondrial ROS scavenger (mitoTEMPO), indicating that ZIKV NS4A induces mitochondrial ROS (**Figure 1F**). These data demonstrate that ZIKV NS4A is responsible for the mitochondrial dysfunction observed following PRV infection of JEG-3 cells.

The role of autophagy in ZIKV-infected placental cells has yet to be studied in depth. Therefore, LC3 was used to monitor autophagy activity in PRV-infected JEG-3 cells. Fluorescence microscopy of LC3-GFP-overexpressing cells revealed presence of LC3-positive puncta in PRV-infected cells when compared to mock-infected cells, and induction or inhibition of autophagy via rapamycin or 3-MA treatment prior to infection either enhanced or suppressed viral replication, respectively (**Supplementary Figures 2A–C**). Next, we observed that overexpression of ZIKV NS4A was sufficient to induce autophagy, as shown from the LC3 puncta formation and increased LC3-II amount, which were both enhanced upon CCCP treatment (**Supplementary Figures 2D, E**). Furthermore, ZIKV NS4A interacted with LC3 upon CCCP-induced activation of mitophagy (**Supplementary Figure 2F**). These data demonstrate that ZIKV NS4A is responsible for PRV-induced autophagy in JEG-3 cells via interaction with LC3.

### 3.2 ZIKV NS4A induces mitophagy

**Figure 2:**
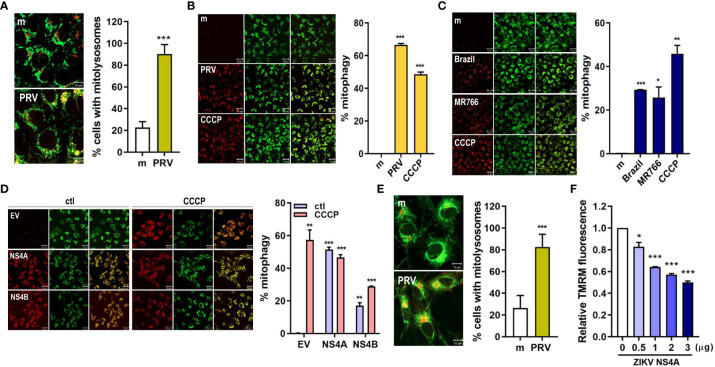
ZIKV NS4A induces mitophagy in human trophoblasts and neural progenitor cells. **(A)** JEG-3 cells were infected with mock- (m) or PRV (MOI 10) and stained with MitoTracker (green) (500 nM) and LysoTracker (red) (75 nM). Scale bar = 10 μm. Mitolysosomes were quantified by measuring percentage of cells with MitoTracker and LysoTracker colocalization (graph). The graph shows mean ± SD (n = 3). Statistical analysis; ***p<0.001, when compared to the mock. **(B-D)** HeLa cells expressing mtKeima (pH-dependent fluorescent protein) were infected with mock (m) or PRV **(B)**, mock (m), Brazil or MR766 **(C)**, or transfected with empty vector (EV), ZIKV NS4A or NS4B **(D)** were treated with CCCP (25 μM) for 2 (h) Green fluorescence of mtKeima reflects mitochondria in the cytosol (green), while red fluorescence of mtKeima reflects mitochondria in lysosomes (red). pH-dependent shift in excitation peaks were quantified to calculate % mitophagy (graph) using Zeiss ZEN software. *p<0.05, **p<0.01, and ***p<0.001, when compared to the mock or EV-transfected control. **(E)** MitoTracker Green and LysoTracker Red staining of mock- vs. PRV-infected human neural progenitor cells (hNPCs). Percentage of cells with MitoTracker and LysoTracker colocalization was calculated (graph). **(F)** TMRM staining of hNPCs transfected with varying concentrations of ZIKV NS4A-encoding plasmid. *p<0.05, ***p<0.001, when compared to the EV-transfected control.

The final step in mitophagy is lysosomal degradation of the damaged, dysfunctional mitochondria via formation of mitolysosomes, which result from fusion between mitophagosome and lysosome. We observed that mitolysosome containing cells increased upon PRV infection of JEG-3 cells, as shown by the increased co-localization of the mitochondria and lysosome (**Figure 2A**). Mitophagy activity can also be measured using HeLa cells stably expressing mtKeima protein with a dual fluorescent tag. The excitation peak of mtKeima protein shifts from green (pH 7) to red (pH 4) depending on the pH, and this shift can be analyzed to measure mitophagy activity in live cells (21). PRV infection of HeLa-mtKeima cells resulted in increased mitophagy activity, which was mimicked in ZIKV NS4A-overexpressing cells (**Figures 2B, D**). Infection with additional strains of ZIKV, Brazil (Asian lineage) and MR766 (African lineage), also resulted in activation of mitophagy, but the levels of activation were not as high as the levels observed in PRV-infected cells (**Figure 2C**). These data demonstrate that PRV-induced mitophagy involves ZIKV NS4A. Mitophagy induction was also observed in mtKeima-expressing JEG-3 cells upon transfection with ZIKV NS4A-encoding plasmid (**Supplementary Figure 3**). In addition, PRV induced formation of mitolysosomes following infection of hNPCs or ZIKV NS4A overexpression resulted in depolarization of MMP, both of which are findings similar to those observed in JEG-3 cells (**Figures 2E, F**). We also confirmed that ZIKV NS4A did not have any effects on the apoptotic signaling pathways in JEG-3 cells, as shown by the lack of enhanced caspase 3 or PARP activation following staurosporine treatment (**Supplementary Figures 4A, B**).

### 3.3 Drp1-mediated mitochondrial fission & PINK1 are important for ZIKV replication

**Figure 3:**
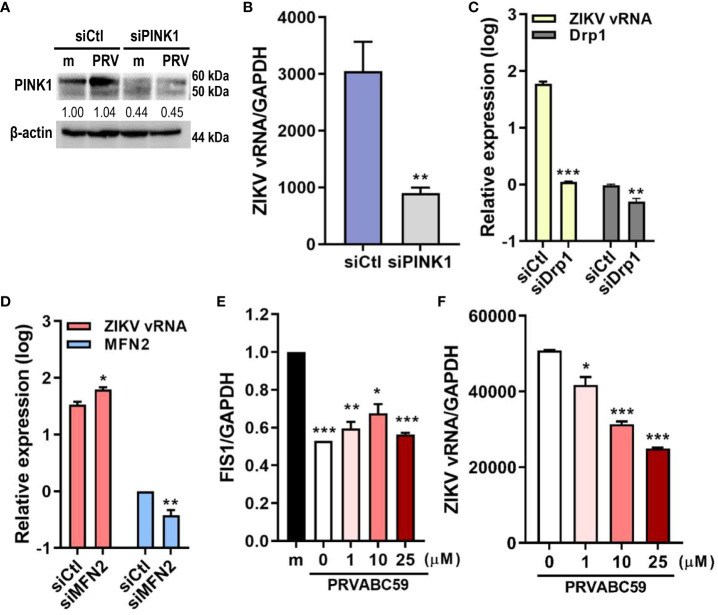
Drp1-mediated mitochondrial fission & PINK1 are important for ZIKV replication. **(A)** Western blot analysis of JEG-3 transfected with ctl (siCtl) vs. PINK1-specific siRNA (siPINK1) to confirm successful knockdown. Band intensities were quantified using ImageJ. **(B)** *ZIKV vRNA* levels were quantified using qRT-PCR in siCtl- vs. siPINK1-transfected cells infected with PRV. **p<0.01, when compared to the siCtl-treated cells **(C, D)** JEG-3 cells transfected with control siRNA (siCtl), Drp1-specific siRNA (siDrp1) **(C)** or MFN2-specific siRNA (siMFN2) **(D)** were infected with PRV (MOI 10). *ZIKV vRNA* expression levels were quantified with qRT-PCR. **(E, F)** Mdivi-1 was added to JEG-3 cells at various concentrations and cells were infected with PRV (MOI 10). Mdivi-1-induced downregulation of mitochondrial fission was confirmed via suppression of *FIS1* mRNA levels **(E)**. Mdivi-1 treatment at various concentrations suppressed *ZIKV vRNA* gene expression levels, as quantified by qRT-PCR **(F)** (mean ± SD; n = 3). *p<0.05, **p<0.01, and ***p<0.001, when compared to the control. Relative expression levels were normalized against GAPDH.

Mitophagy activation can occur via ubiquitin (PINK1/Parkin)-dependent or ubiquitin-independent pathways. We determined if PRV utilized PINK1/Parkin signaling to activate mitophagy during JEG-3 infection. We observed that suppression of PINK1 expression via siRNA transfection resulted in lower *ZIKV vRNA* levels when compared to negative control siRNA-transfected cells (**Figures 3A, B**). Meanwhile, the disruption of mitochondrial dynamics via knockdown of Drp1, a key regulator of mitochondrial dynamics, suppressed ZIKV replication, while knockdown of fusion protein, MFN2, enhanced viral replication (**Figures 3C, D**). Mdivi-1, which inhibits Drp1 and promotes mitochondrial fusion, treatment prior to PRV infection also suppressed both FIS1, a mitochondrial fission protein that interacts with Drp1, and ZIKV vRNA levels (**Figures 3E, F**). These findings demonstrate that Drp1-mediated mitochondrial fission and PINK1 are important regulators of ZIKV replication in JEG-3 cells.

### 3.4 Mitophagy receptors BNIP3 and NIX modulate ZIKV replication

**Figure 4:**
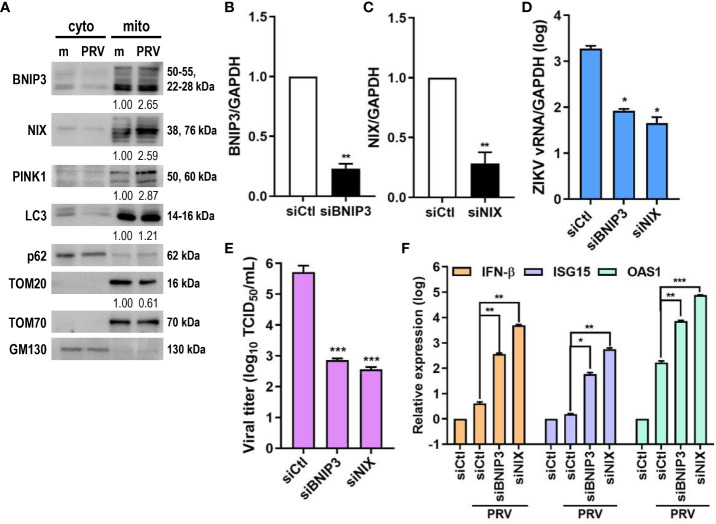
ZIKV NS4A upregulates mitophagy receptor expression, and BNIP3 and NIX modulate ZIKV replication. **(A)** Western blot analysis of mitophagy receptor expression levels in cytosolic (cyto) and mitochondrial (mito) fractions obtained from mock- vs. PRV-infected JEG-3 cells. Band intensities were quantified using ImageJ and normalized against TOM70. **(B, C)** JEG-3 cells were transfected with siCtl, siBNIP3 **(B)** or siNIX **(C)** and infected with PRV (MOI 10). *BNIP3* and *NIX* mRNA levels were determined by qRT-PCR. Data represented as mean ± SD. **p<0.01, when compared to the siCtl-transfected cells. Relative expression levels were normalized against GAPDH. **(D, F)** qRT-PCR analysis of BNIP3 or NIX siRNA-transfected JEG-3 cells infected with PRV (MOI 10). Relative viral transcription levels **(D)** and *IFN-β, ISG15*, and *OAS1* mRNA levels **(F)** were determined. Relative expression levels were normalized against GAPDH. **(E)** TCID_50_ assay of Vero cells infected with supernatants collected at 48 hpi from siBNIP3- or siNIX-transfected JEG-3 cells infected with PRV (MOI 10). Data represented as mean ± SD. *p<0.05, **p<0.01, and ***p<0.001, when compared to the siCtl-treated PRV-infected cells.

Ubiquitin-independent activation of mitophagy involves various mitophagy receptors, including BNIP3 and NIX (25). We investigated whether PRV-induced mitophagy in JEG-3 cells also relied on PINK1/Parkin-independent pathway. Cytosolic and mitochondrial fractions of mock- vs. PRV-infected JEG-3 cells were analyzed for mitophagy receptor expression levels. ZIKV infection upregulated mitochondrial expression levels of BNIP3, NIX, and PINK1 (**Figure 4A**). Tom70, which is anchored to the mitochondrial outer membrane, was used as the mitochondrial marker for protein normalization. Next, we determined whether BNIP3 and NIX were capable of modulating ZIKV replication. Upon knockdown of BNIP3 or NIX, *ZIKV vRNA* levels were downregulated in PRV-infected cells (**Figures 4B–D**). Furthermore, the knockdown of BNIP3 or NIX expression prior to PRV infection resulted in reduced viral infectivity, as shown by reduced viral titers (**Figure 4E**). We observed that in absence of BNIP3 or NIX expression, *IFN-β, ISG-15*, and *OAS1* mRNA levels were upregulated, suggesting that PRV-induced mitophagy downregulates type I IFN response (**Figure 4F**). These results indicate that PRV-induced mitophagy occurs in both ubiquitin-dependent and -independent pathways and plays a pro-viral function to facilitate ZIKV replication and suppress antiviral response in JEG-3 cells.

### 3.5 ZIKV NS4A inhibits MAVS

**Figure 5:**
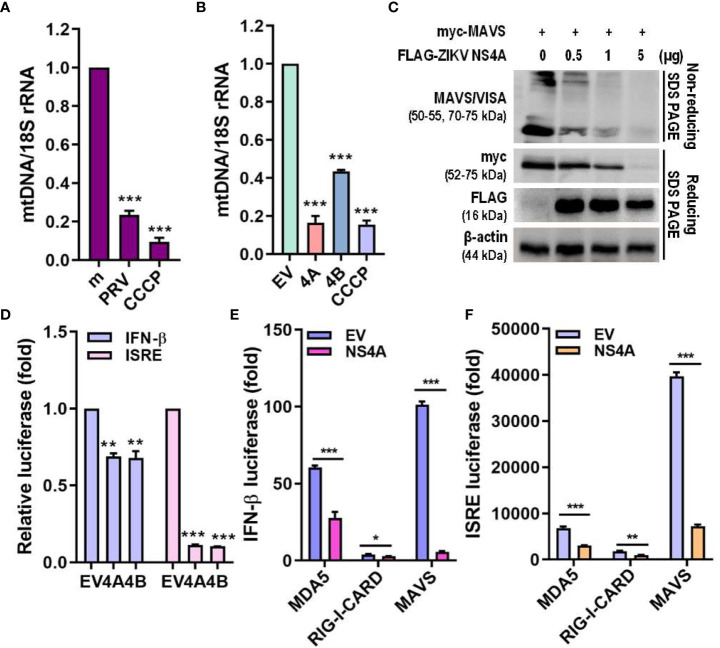
ZIKV NS4A reduces mitochondrial mass & blocks MAVS oligomerization to suppress IFN response. **(A, B)** Genomic DNA isolated to determine relative mitochondrial DNA (mtDNA) present in JEG-3 cells infected with mock (m), PRV (MOI 10) or treated with CCCP (25 μM, 2 h) **(A)** or transfected with empty vector (EV), ZIKV NS4A, NS4B or treated with CCCP (25 μM, 2 h) **(B)**. Relative mtDNA amount was normalized against 18S rRNA. *p<0.05, **p<0.01, and ***p<0.001, when compared to the m or EV control. **(C)** Western blot analysis of MAVS oligomerization and expression levels in JEG-3 cells transfected with MAVS in combination with increasing concentrations of ZIKV NS4A. **(D)** JEG-3 cells overexpressing IFN-β or ISRE reporter plasmids were transfected with EV, ZIKV NS4A or NS4B and treated with poly(I:C) (20 μg/mL, 2h). Luciferase reporter assay is shown (mean ± SD). **(E, F)** Luciferase assay of HEK293T cells transfected with IFN-β or ISRE reporter plasmids in combination with ZIKV NS4A and one of the following RLR signaling effectors (MDA5, RIG-I, MAVS, TBK1, IRF3). Data represented as mean ± SD. *p<0.05, **p<0.01, and ***p<0.001, when compared to the EV control.

Mitophagy results in degradation of the mitochondria, resulting in reduced mitochondrial mass and decreased mitochondrial components, including mtDNA. Both PRV infection and ZIKV NS4A overexpression reduced the amount of mtDNA (**Figures 5A, B**). MAVS localization to the mitochondria is required for activation of downstream RLR signaling pathway, which subsequently promotes type I IFN and proinflammatory cytokine expression (26). Therefore, the effect of ZIKV NS4A on MAVS-mediated antiviral signaling was investigated. Increasing concentrations of ZIKV NS4A resulted in inhibition of MAVS expression and oligomerization (**Figure 5C**). Luciferase reporter assays also revealed that upon poly(I:C) treatment, both ZIKV NS4A and NS4B suppressed IFN-β and ISRE promoter activities (**Figure 5D**). To determine which RLR signaling effector ZIKV NS4A interacts with to suppress type I IFN signaling, luciferase reporter assays were performed with MDA5, RIG-I, MAVS-encoding plasmids (**Figures 5E, F**). Results showed that ZIKV NS4A effectively inhibited MDA5, RIG-I, or MAVS-mediated IFN-β and ISRE promoter activities. Altogether, these findings suggest that ZIKV NS4A-induced mitophagy degrades mitochondrial components like mtDNA and MAVS, and ZIKV NS4A-induced suppression of IFN response occurs through inhibition of MAVS expression and oligomerization.

### 3.6 ZIKV-infected sEVs contain mitochondrial components

**Figure 6:**
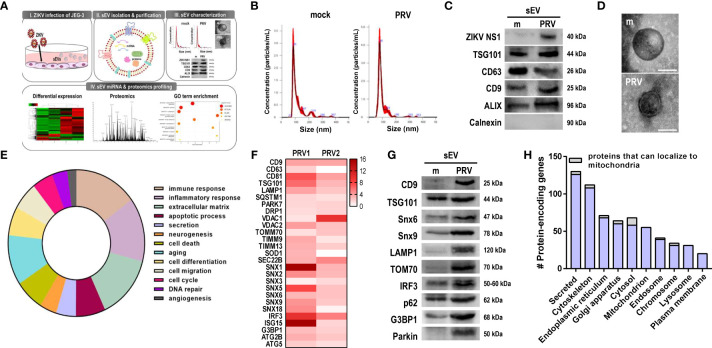
Small extracellular vesicles (sEV) proteomics profiling revealed presence of mitochondrial components. **(A)** Schematic representation of the experimental procedures taken to isolate, purify, and characterize sEVs released from mock- vs. PRV-infected JEG-3 cells. Liquid chromatography with tandem mass spectrometry (LC–MS/MS) analysis was performed to characterize the contents of sEVs from PRV-infected JEG-3 cells **(B)** Nanoparticle tracking analysis of sEVs secreted by mock- or PRV-infected JEG3 cells is shown. **(C)** Western blot analysis of sEVs confirm the presence of ZIKV NS1 and sEV markers (CD9, CD63, TSG101, and ALIX). Endoplasmic reticulum localized calnexin was used as a negative marker. **(D)** Transmission electron microscopy of sEVs. Scale bar = 100 nm. **(E)** Pie chart of functional Gene Ontology (GO) terms associated with differentially expressed proteins detected within sEVs (PRV- vs. mock-infected; p < 0.05, fold change > 2). **(F)** Heatmap of top expressed proteins that were upregulated in PRV-infected sEVs. Two biological replicates per samples were included for further analysis. **(G)** LC–MS/MS-based proteomics data were validated by Western blot analysis. Snx6, Snx9, LAMP1, TOM70, IRF3, p62, and G3BP1 were upregulated in PRV-infected sEVs compared with mock sEVs. **(H)** Number of protein-encoding genes of the detected proteins were quantified according to their subcellular localization. Proteins that can localize to the mitochondria are shown in gray.

To investigate the effect of ZIKV infection on sEV protein composition, conditioned medium of PRV-infected JEG-3 cells were collected for sEV isolation and characterization (**Figure 6A**). Mean size and concentration of extracellular vesicles secreted by mock- vs. PRV-infected JEG-3 cells were analyzed by nanoparticle tracking analysis, which showed that majority of the EVs isolated were under 150 nm (mock-infected: 119.8 nm; PRV-infected: 96.8 nm) (**Figure 6B**). Interestingly, PRV-infected JEG-3 cells secreted less EVs (2.52 x 10^10^ particles/mL) when compared to mock-infected cells (1.92 x 10^11^ particles/mL). Western blot analysis revealed that sEVs expressed common EV markers like CD63, CD9, TSG101, and ALIX, which are involved in sEV biogenesis or trafficking, and did not express calnexin, an ER marker that was used as a negative control (**Figure 6C**) (27). Interestingly, the infected sEVs also contained ZIKV NS1, which is in line with previous report on presence of NS1 dimers on surface of exosome-like EVs (28). Further characterization of sEV morphology was analyzed by TEM that revealed the presence of small, double-membraned nanovesicles (**Figure 6D**).

After confirming the successful isolation of sEVs, LC-MS/MS-based proteomics profiling was performed to investigate the protein components of sEVs secreted by PRV- vs. mock-infected JEG-3 cells, and samples were prepared in two biological replicates. Differential expression profiles of the protein-encoding genes were similar between mock-infected and PRV-infected sEVs (**Supplementary Figure 5A**). Total of 2301 proteins were detected, and 329 proteins were differentially regulated (p-value <0.05, fold change > 2) in sEVs during PRV infection of JEG-3 cells. Gene ontology pathway analysis revealed that large proportions of the protein-encoding genes of the proteins differentially regulated during infection were involved in immune response or inflammatory response (**Figure 6E**). Differential expression levels of the upregulated proteins of interest were represented as a heatmap (**Figure 6F**). Interestingly, PRV infection induced upregulation of sEV biomarkers including tetraspanins (CD9 and CD63) and endosomal sorting complexes required for transport (ESCRT) protein TSG101. Additionally, SEC22B, which is known for its function during endoplasmic reticulum-to-Golgi trafficking and recently reported to be present on autophagic intermediates destined for secretion, was found to be upregulated (29). Several proteins of the sorting nexin (Snx) family were also significantly upregulated in PRV-infected sEVs.

Proteomics analysis of PRV-infected sEVs also revealed the presence of mitochondrial proteins, such as VDAC1, VDAC2, TIMM9, TIMM13, TOM70, and Parkin. To evaluate the correlation between quantitative proteomics results, western blot analysis was performed and we found upregulation of the following PRV-infected sEV cargo, CD9, TSG101, Snx6 and Snx9, LAMP1, TOM70, IRF3, p62, G3BP1, and Parkin when compared to mock-infected sEVs (**Figure 6G**). Proteins were also analyzed according to their subcellular localization. The highest number of proteins detected within the sEVs were secreted, and proteins that localized to the mitochondria were mostly upregulated in PRV-infected sEVs (**Figure 6H** and **Supplementary Figures 5B–D**). These findings highlight the potential of the mitochondria as a source of sEVs secreted during PRV infection by JEG-3 cells, and the possible role of Snx6 during MDV formation and biogenesis.

In addition, small RNA sequencing of the miRNA content encapsulated within sEVs revealed that ZIKV differentially regulated expression levels of extracellular miRNAs during infection. Analysis of the sequencing data revealed similarities in miRNA expression profiles between sEV samples isolated from mock- vs. PRV-infected JEG-3 cells (**Supplementary Figures 6A, B**) and miR-30c, -15a, and -204 were among the top 5 miRNAs that were significantly upregulated upon PRV infection. Small RNA-sequencing of sEVs secreted by ZIKV-infected JEG-3 revealed that ZIKV differentially regulated miRNAs involved in cellular pathways like immune response, inflammation, secretion, and autophagy.

**Figure 7:**
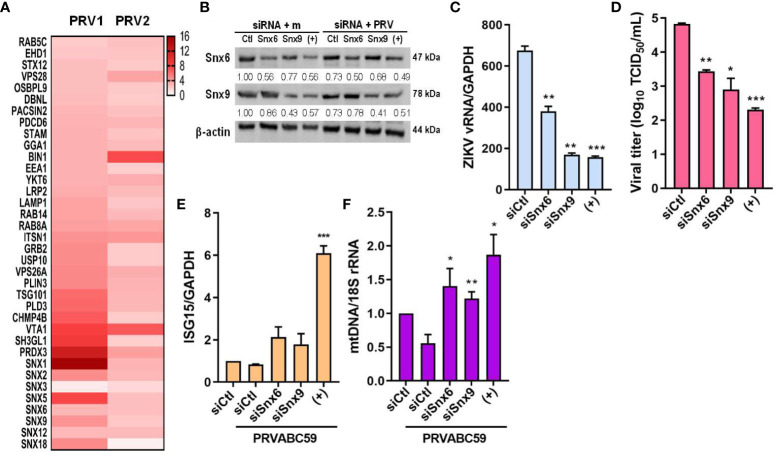
MDV components (Snx6 and Snx9) regulate ZIKV replication & infectivity. **(A)** Heatmap of upregulated sEV proteins that can localize to the endosome. Snx6 and 9 were chosen for further analysis. **(B)** Western blot analysis of JEG-3 cells transfected with Snx6- or Snx9-specific siRNAs and infected with mock or PRV (MOI 10). Snx6 and Snx9 protein levels were determined and quantified below the blot. **(C)** qRT-PCR quantification of *ZIKV vRNA* transcription levels in siSnx6- and/or siSnx9-transfected JEG-3 cells infected with PRVABC59. Expression levels were quantified relative to siCtl-transfected JEG-3 cells infected with PRV. **(D)** TCID_50_ assay of Vero cells infected with supernatants collected at 48 hpi from siSnx6- and/or siSnx9-transfected JEG-3 cells infected with PRV. **(E)** siSnx6- and/or siSnx9-transfected JEG-3 cells were infected with PRV for 24h. Relative *ISG15* mRNA levels were quantified relative to siCtl-transfected JEG-3 cells infected with PRV. **(F)** Cytosolic mtDNA amount was quantified with qRT-PCR. (+) = JEG-3 cells transfected with siSnx6 and siSnx9 in combination. Data represented as mean ± SD (n=3). *p<0.05, **p<0.01, and ***p<0.001, when compared to the siCtl-transfected cells infected with PRV.

Next, we found that sEVs derived from PRV-infected JEG-3 cells contained proteins that localize to the endosome, and many proteins belonging to the sorting nexin (Snx) family were detected, including Snx1, 5, 6, and 9 (**Figure 7A**). Snx9 has been reported to regulate loading of mitochondrial proteins into MDVs, which can be secreted as sEVs or target mitochondrial components for lysosomal degradation (30). Therefore, we further investigated the importance of MDVs during PRV infection in JEG-3 cells via Snx6- and Snx9-specific siRNA transfection individually or in combination prior to infection. Snx6 belongs to the same family of proteins as Snx9, which is required for formation of MDVs targeted for extracellular release or lysosomal degradation (31). Since Snx6 has not been associated with any functional roles during MDV biogenesis and trafficking, the effect of Snx6 and Snx9 knockdown on PRV viral replication and infectivity was analyzed. Knockdown of Snx6 and/or Snx9 downregulated *ZIKV vRNA* levels, in addition to reduced viral titers (**Figures 7B–D**). Increased mRNA expression levels of *ISG15* upon Snx6 and/or Snx9 knockdown during ZIKV infection were observed, suggesting that disruption of MDV formation during ZIKV infection affects the host antiviral response in JEG-3 cells (**Figure 7E**). MDVs have also been reported to contain mtDNA, which can act as DAMPs to activate inflammatory or immune responses when present in the cytosol (30). Knockdown of Snx6 and/or Snx9 increased knockdown of Snx6 and/or Snx9 increased the amount of cytosolic mtDNA when compared to siCtl-transfected cells that were infected with PRV (**Figure 7F**). These data provide the possibility that PRV hijacks mitochondrial quality control at the level of MDV formation and secretion to facilitate viral replication in placental cells.

## 4 Discussion

**Figure 8:**
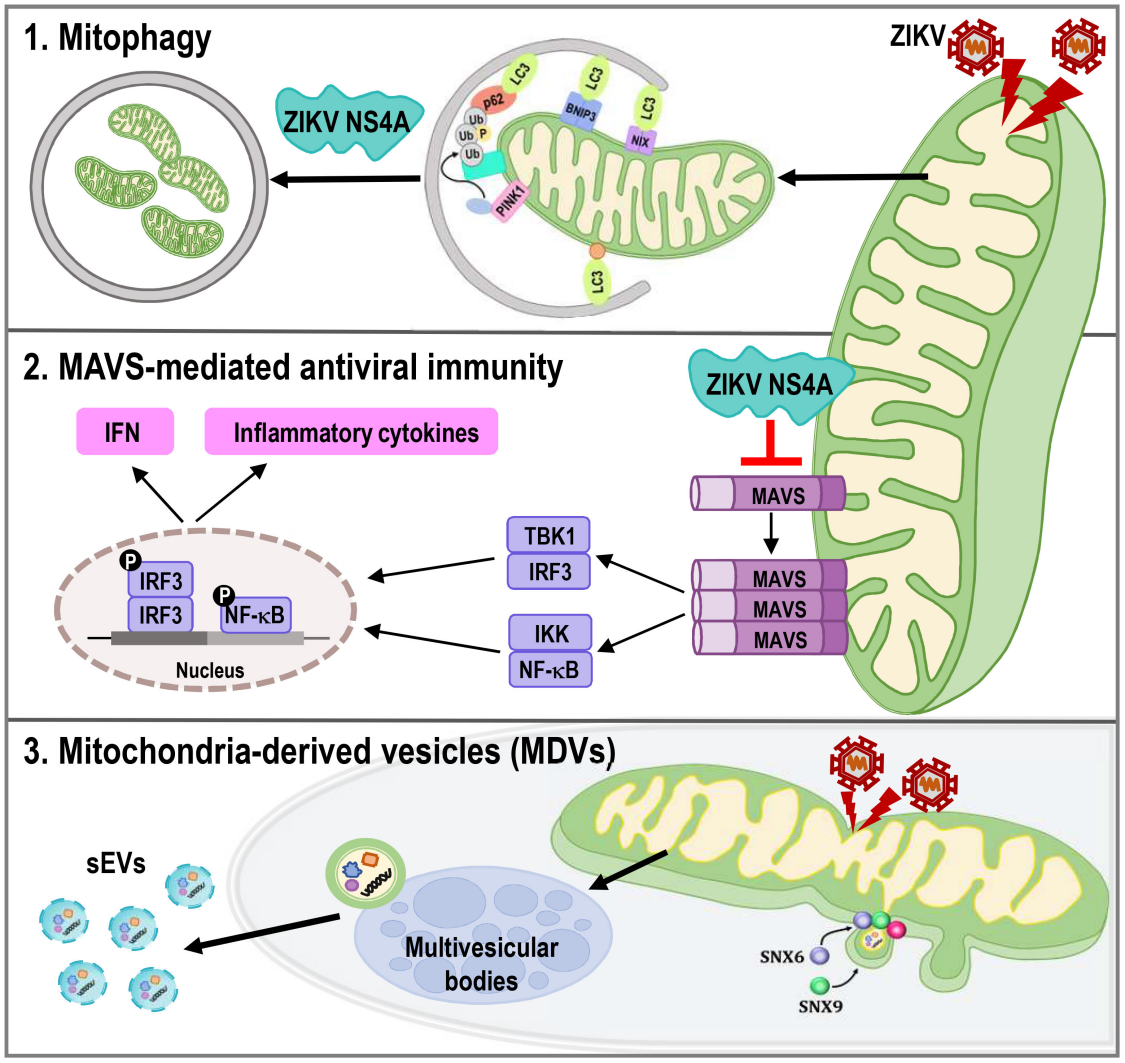
ZIKV NS4A dysregulates mitochondrial dynamics and quality control mechanisms to suppress host antiviral response in trophoblast cells. ZIKV NS4A triggers changes in mitophagy, and MAVS-mediated antiviral immunity to evade the host innate immune response and facilitate viral infection, while ZIKV modulation of MDV components, such as Snx6 and Snx9, may affect ZIKV replication and infectivity.

Mitochondria are highly dynamics organelles undergoing constant changes in numbers and activities in response to various physiological or pathological conditions. Mitophagy and recurrent cycles of fission and fusion are established mechanisms for ensuring mitochondrial quality, while MDVs, which are derived from the mitochondrial surface and directed to lysosomes or peroxisomes for degradation, have emerged as relatively new mechanism. Here we report that ZIKV triggers changes in mitochondrial dynamics, mitophagy, and MDVs to evade the host innate immune response and facilitate viral infection (**Figure 8**).

Mitochondrial dynamics and mitophagy coordinate with each other to maintain mitochondrial function, integrity, and quantity (32). Mitochondrial fission precedes mitophagy to separate dysfunctional, depolarized mitochondria from the rest of the mitochondrial network for engulfment by autophagosomes, and inhibition of fission results in decreased mitophagy levels (33). Our findings demonstrate that PRV induces mitophagy in JEG-3 cells upon infection, and Drp1-mediated fission and PINK1 were shown to be important modulators of ZIKV viral replication (**Figures 2**, **3**).

Mitochondrial fusion is important for MAVS to interact with downstream signaling molecules and maintain the MMP, while mitochondrial fission results in smaller mitochondrial size and diminished RLR signaling (4, 34–37). Mitochondrial surface provides a platform for MAVS-mediated antiviral signaling, and ZIKV NS4A has been associated with inhibition of type I IFN response via suppression of RLR-MAVS interaction (7). In line with previous reports, our data show that ZIKV NS4A reduced mitochondrial mass via mitophagy induction to suppress MAVS-mediated type I IFN production in JEG-3 cells, as shown by the reduction of mtDNA, inhibition of MAVS expression and oligomerization, and subsequent suppression of type I IFN (**Figure 5**). Recently, Shah et al. revealed that ZIKV NS4A interacts with many host proteins involved in mitochondrial organization, trafficking, oxidative respiration, autophagy and immunity by comparing the flavivirus-human networks. In addition, ANKLE2 was identified as a key molecule which can contribute to neuroblast development and microcephaly in humans (38). Given the possible role of mitophagy in infected hNPCs in causing neurodevelopmental disorders like microcephaly, it will be interesting to further investigate how ZIKV NS4A manipulates mitochondrial quality control mechanisms to enhance viral infection and evade neural cell differentiation.

Several RNA viruses have been reported to impact various processes of mitochondrial dynamics and quality control to suppress the host antiviral innate immune response and enhance viral replication (39–41). For example, Dengue virus (DENV) alters mitochondrial dynamics to increase antiviral immune evasion. Previous studies have demonstrated the beneficial role of mitochondrial fusion during DENV replication, and induction of mitochondrial fragmentation or fission via CCCP treatment or Drp1 knockdown resulted in impaired viral replication (42, 43). In correlation with this study, our previous reports suggest the mitochondrial elongation during ZIKV infection in human umbilical cord mesenchymal stem cells (20). On the contrary, PRVABC59 infection in JEG-3 cells induces mitochondrial fission and ZIKV NS4A was shown to disrupt mitochondrial dynamics and mitochondrial respiration profile, in addition to depolarization of mitochondrial membrane potential and mitochondrial ROS production, all of which are cellular events observed during mitophagy (**Figures 1** and **Supplementary Figure 1**). It is highly likely that mitochondria morphodynamics depends on the cell type and viruses, but regardless of viruses, mitochondrial perturbation can protect viruses from antiviral IFN response and create a favorable replicative environment.

The flaviviral RNA genome encodes 7 nonstructural proteins (NSs) that have essential functions in viral replication and immune evasion, and NS4A, NS4B, and NS5 have been reported to inhibit JAK-STAT and RLR signaling through various mechanisms to enhance viral pathogenesis (44–48). NS5A of Hepatitis C virus (HCV), also belonging to the *Flaviviridae* family, upregulates mitochondrial fission and mitophagy to block HCV-induced mitochondrial damage from spreading and triggering apoptosis, and maintain a cellular environment that is beneficial for viral replication and dissemination (49, 50). ZIKV NS4A and NS4B inhibit neurogenesis via induction of autophagy in NSCs via inhibition of Akt/mTOR pathway (8). Dengue virus (DENV) NS4A promotes viral replication complex formation and is sufficient to induce autophagy, while Japanese encephalitis virus (JEV) NS4A localizes to the mitochondria to interact with PINK1 to dysregulate mitochondrial quality control by inducing mitophagy (41, 51, 52). According to Ponia et al, ZIKV NS5 suppressed mitophagy by inhibiting mitochondrial translocation of Ajuba and inducing early expression of proinflammatory chemokines to enhance tissue dissemination (53). However, modulation of autophagic or mitophagic processes during ZIKV infection of placental trophoblast cells have yet to be studied. In this study, we observed that ZIKV NS4A localized to the mitochondria and interacted with LC3 in CCCP-treated JEG-3 cells to induce mitophagy upon PRV infection **(** **Figures 1**, **2**, and **Supplementary Figure 2**). ZIKV NS4A overexpression alone upregulated expression levels of mitophagy receptors like BNIP3, NIX, and PINK1, and suppression of BNIP3- or NIX-mediated mitophagy resulted in enhanced *IFN-β*, *ISG15*, and *OAS1* levels (**Figure 4**).

Analysis of the secretory profiles of JEG-3 cells during ZIKV infection, in terms of sEV miRNA and protein composition, allows identification of novel, signature secretory profiles of ZIKV-infected trophoblast cells and provide additional biomarkers that can be used in the development of noninvasive diagnostic methods that can detect ZIKV during pregnancy. Small RNA sequencing analysis revealed miR-15a, -30c, and -204a to be among the top 5 most upregulated miRNAs in PRV-infected sEVs released from JEG-3 cells. miR-30c, the most significantly upregulated miRNA within PRV-infected sEVs, inhibits mitochondrial fission via p53-mediated suppression of Drp1 (54). Proteomics profiling of sEVs revealed presence of mitochondrial proteins, including TOM70, VDAC1, and MDV-associated Snx9, which were mostly upregulated in ZIKV-infected sEVs (**Figure 6** and **Supplementary Figure 5**). TOM70 was previously reported to interact with MAVS during RNA virus infection and function as an adaptor protein linking MAVS and TBK1/IRF3 (55). Secretion of TOM70 via sEVs may suggest another immune evasion mechanism for ZIKV to suppress activation of type I IFN response mediated via MAVS. In addition, the presence of ZIKV NS1 in infected sEVs released by JEG-3 cells further highlights the importance of nonstructural proteins during ZIKV pathogenesis.

Several proteins of the Snx family were also upregulated in PRV-infected sEVs. Snx proteins are involved in endocytosis and protein trafficking (31). Snx5, for example, has been reported to mediate virus-induced autophagy, and knockdown of Snx5 resulted in decreased autophagy induction by ZIKV in Dong et al. (56). Upregulation of Snx5 in PRV-infected sEVs suggest the possible role of secretory autophagy during infection of placental cells. Snx1 was also observed to be differentially upregulated in EVs secreted by ZIKV-inoculated trophoblast cells in a macaque ZIKV infection model of pregnancy (16). In addition to Snx9, various proteins of the Snx family were detected to be upregulated in ZIKV-infected sEVs, and further analysis of Snx6 and 9 showed that disruption of MDV biogenesis affects ZIKV replication and infectivity, in addition to increasing the amount of cytosolic mtDNA following infection (**Figure 7**). According to our data, Snx6, similar to Snx9, may also function as a regulator of MDV formation. Mitophagy can also occur via MDVs to remove parts of the mitochondria through a process known as micromitophagy, and modulation of MDV components may affect ZIKV replication and infectivity by disrupting ZIKV-induced mitophagy (57).

Currently, ZIKV-specific therapeutic treatments or vaccines have yet to be clinically approved. Because sEVs are capable of crossing blood-tissue barriers, recent strategies for antiviral therapy against neurotropic viruses include targeted delivery of sEVs containing antiviral candidate drugs, peptides, or siRNAs to the brain. EVs containing IFITM3 were able to be delivered from pregnant mice to the fetuses and suppress ZIKV infection in both mothers and fetuses, while sEVs that overexpress rabies virus glycoprotein were capable of transplacental delivery of anti-ZIKV siRNA to the fetus brain in mice (58, 59). Our study uncovered molecular mechanisms underlying ZIKV-mediated manipulation of mitochondrial quality control and identified important regulators of ZIKV-induced mitophagy or MDV formation that may lead to the discovery of novel therapeutic targets that can be loaded into sEVs for targeted delivery. It will be interesting to carry out further studies on the potential antiviral effects of sEVs carrying siRNAs against MDV or mitophagy receptors, or the possibility of controlling congenital ZIKV infections via inhibition of mitophagy or MDV/sEV biogenesis and secretion.

## Data availability statement

The datasets presented in this study can be found in online repositories. The names of the repository/repositories and accession number(s) can be found below: PXD041807 (ProteomeXchange Consortium via the PRIDE) and GSE228310 (GEO).

## Author contributions

Conceptualization, JKL, OSS. Methodology JKL. Formal analysis, JKL. Investigation, JKL, OSS writing—original draft preparation, JKL. Writing—review and editing, OSS. Visualization, OSS. Supervision, OSS. Project administration, JKL. Funding acquisition, OSS. All authors have read and agreed to the published version of the manuscript. All authors contributed to the article and approved the submitted version.

## References

[1] De Araújo TVB De Alencar Ximenes RA De Barros MIranda-Filho D Souza WV Montarroyos UR De Melo APL. Association between microcephaly, Zika virus infection, and other risk factors in Brazil: final report of a case-control study. Lancet Infect Dis (2018) 18:328–36. doi: 10.1016/S1473-3099(17)30727-2 PMC7617036 29242091

[2] Pierson TC Diamond MS. The emergence of Zika virus and its new clinical syndromes. Nature (2018) 560:573–81. doi: 10.1038/s41586-018-0446-y 30158602

[3] García CC Vázquez CA Giovannoni F Russo CA Cordo SM Alaimo A. Cellular organelles reorganization during zika virus infection of human cells. Front Microbiol (2020) 11:1558. doi: 10.3389/fmicb.2020.01558 32774331 PMC7381349

[4] Castanier C Garcin D Vazquez A Arnoult D. Mitochondrial dynamics regulate the RIG-I-like receptor antiviral pathway. EMBO Rep (2010) 11:133–8. doi: 10.1038/embor.2009.258 PMC2828750 20019757

[5] Soubannier V Mclelland G-L Zunino R Braschi E Rippstein P Fon EA. A vesicular transport pathway shuttles cargo from mitochondria to lysosomes. Curr Biol (2012) 22:135–41. doi: 10.1016/j.cub.2011.11.057 22226745

[6] Sugiura A Mclelland GL Fon EA Mcbride HM. A new pathway for mitochondrial quality control: mitochondrial-derived vesicles. EMBO J (2014) 33:2142–56. doi: 10.15252/embj.201488104 PMC4282503 25107473

[7] Ma J Ketkar H Geng T Lo E Wang L Xi J. Zika virus non-structural protein 4A blocks the RLR-MAVS signaling. Front Microbiol (2018) 9:1350. doi: 10.3389/fmicb.2018.01350 29988497 PMC6026624

[8] Liang Q Luo Z Zeng J Chen W Foo S-S Lee S-A. Zika virus NS4A and NS4B proteins deregulate Akt-mTOR signaling in human fetal neural stem cells to inhibit neurogenesis and induce autophagy. Cell Stem Cell (2016) 19:663–71. doi: 10.1016/j.stem.2016.07.019 PMC5144538 27524440

[9] Yang S Gorshkov K Lee EM Xu M Cheng Y-S Sun N. Zika virus-induced neuronal apoptosis via increased mitochondrial fragmentation. Front Microbiol (2020) 11:598203. doi: 10.3389/fmicb.2020.598203 33424801 PMC7785723

[10] Link N Chung H Jolly A Withers M Tepe B Arenkiel BR. Mutations in ANKLE2, a ZIKA virus target, disrupt an asymmetric cell division pathway in Drosophila neuroblasts to cause microcephaly. Dev Cell (2019) 51:713–729.e716. doi: 10.1016/j.devcel.2019.10.009 31735666 PMC6917859

[11] Lal CV Olave N Travers C Rezonzew G Dolma K Simpson A. Exosomal microRNA predicts and protects against severe bronchopulmonary dysplasia in extremely premature infants. JCI Insight (2018) 3. doi: 10.1172/jci.insight.93994 PMC5922295 29515035

[12] Phipps EA Thadhani R Benzing T Karumanchi SA. Pre-eclampsia: pathogenesis, novel diagnostics and therapies. Nat Rev Nephrol (2019) 15:275–89. doi: 10.1038/s41581-019-0119-6 PMC6472952 30792480

[13] Chahar HS Bao X Casola A. Exosomes and their role in the life cycle and pathogenesis of RNA viruses. Viruses (2015) 7:3204–25. doi: 10.3390/v7062770 PMC4488737 26102580

[14] Théry C Witwer KW Aikawa E Alcaraz MJ Anderson JD Andriantsitohaina R. Minimal information for studies of extracellular vesicles 2018 (MISEV2018): a position statement of the International Society for Extracellular Vesicles and update of the MISEV2014 guidelines. J Extracellular Vesicles (2018) 7:1535750. doi: 10.1080/20013078.2018.1535750 30637094 PMC6322352

[15] Bai K Li X Zhong J Ng EH Yeung WS Lee C-L. Placenta-derived exosomes as a modulator in maternal immune tolerance during pregnancy. Front Immunol (2021) 12:671093. doi: 10.3389/fimmu.2021.671093 34046039 PMC8144714

[16] Block LN Schmidt JK Keuler NS Mckeon MC Bowman BD Wiepz GJ. Zika virus impacts extracellular vesicle composition and cellular gene expression in macaque early gestation trophoblasts. Sci Rep (2022) 12:7348. doi: 10.1038/s41598-022-11275-9 35513694 PMC9072346

[17] Kim JA Seong RK Kumar M Shin OS. Favipiravir and ribavirin inhibit replication of Asian and African strains of Zika virus in different cell models. Viruses (2018) 10. doi: 10.3390/v10020072 PMC5850379 29425176

[18] Kim JA Seong RK Son SW Shin OS. Insights into ZIKV-mediated innate immune responses in human dermal fibroblasts and epidermal keratinocytes. J Invest Dermatol (2019) 139:391–9. doi: 10.1016/j.jid.2018.07.038 30218650

[19] Seong RK Lee JK Shin OS. Zika virus-induction of the suppressor of cytokine signaling 1/3 contributes to the modulation of viral replication. Pathogens (2020) 9. doi: 10.3390/pathogens9030163 PMC7157194 32120897

[20] Seong RK Lee JK Cho GJ Kumar M Shin OS. mRNA and miRNA profiling of Zika virus-infected human umbilical cord mesenchymal stem cells identifies miR-142-5p as an antiviral factor. Emerg Microbes Infect (2020) 9:2061–75. doi: 10.1080/22221751.2020.1821581 PMC7534337 32902370

[21] Um J-H Kim YY Finkel T Yun J. Sensitive measurement of mitophagy by flow cytometry using the pH-dependent fluorescent reporter mt-Keima. JoVE (Journal Visualized Experiments) (2018):e58099. doi: 10.3791/58099 PMC6126785 30148491

[22] Kim AJ Jee HJ Song N Kim M Jeong SY Yun J. p21(WAF(1)/C(1)P(1)) deficiency induces mitochondrial dysfunction in HCT116 colon cancer cells. Biochem Biophys Res Commun (2013) 430:653–8. doi: 10.1016/j.bbrc.2012.11.096 23211592

[23] Taguchi N Ishihara N Jofuku A Oka T Mihara K. Mitotic phosphorylation of dynamin-related GTPase Drp1 participates in mitochondrial fission. J Biol Chem (2007) 282:11521–9. doi: 10.1074/jbc.M607279200 17301055

[24] Foo J Bellot G Pervaiz S Alonso S. Mitochondria-mediated oxidative stress during viral infection. Trends Microbiol (2022) 30:679–92. doi: 10.1016/j.tim.2021.12.011 35063304

[25] Onishi M Yamano K Sato M Matsuda N Okamoto K. Molecular mechanisms and physiological functions of mitophagy. EMBO J (2021) 40:e104705. doi: 10.15252/embj.2020104705 33438778 PMC7849173

[26] Seth RB Sun L Ea C-K Chen ZJ. Identification and characterization of MAVS, a mitochondrial antiviral signaling protein that activates NF-κB and IRF3. Cell (2005) 122:669–82. doi: 10.1016/j.cell.2005.08.012 16125763

[27] Lötvall J Hill AF Hochberg F Buzás EI Di Vizio D Gardiner C. Minimal experimental requirements for definition of extracellular vesicles and their functions: a position statement from the International Society for Extracellular Vesicles. J Extracellular Vesicles (2014) 3:26913. doi: 10.3402/jev.v3.26913 25536934 PMC4275645

[28] Safadi DE Lebeau G Lagrave A Mélade J Grondin L Rosanaly S. Extracellular vesicles are conveyors of the NS1 toxin during dengue virus and Zika virus infection. Viruses (2023) 15:364. doi: 10.3390/v15020364 36851578 PMC9965858

[29] Kimura T Jia J Claude-Taupin A Kumar S Choi SW Gu Y. Cellular and molecular mechanism for secretory autophagy. Autophagy (2017) 13:1084–5. doi: 10.1080/15548627.2017.1307486 PMC5486376 28368721

[30] Todkar K Chikhi L Desjardins V El-Mortada F Pépin G Germain M. Selective packaging of mitochondrial proteins into extracellular vesicles prevents the release of mitochondrial DAMPs. Nat Commun (2021) 12:1–12. doi: 10.1038/s41467-021-21984-w 33785738 PMC8009912

[31] Worby CA Dixon JE. Sorting out the cellular functions of sorting nexins. Nat Rev Mol Cell Biol (2002) 3:919–31. doi: 10.1038/nrm974 12461558

[32] Pickles S Vigié P Youle RJ. Mitophagy and quality control mechanisms in mitochondrial maintenance. Curr Biol (2018) 28:R170–85. doi: 10.1016/j.cub.2018.01.004 PMC7255410 29462587

[33] Twig G Elorza A Molina AJ Mohamed H Wikstrom JD Walzer G. Fission and selective fusion govern mitochondrial segregation and elimination by autophagy. EMBO J (2008) 27:433–46. doi: 10.1038/sj.emboj.7601963 PMC2234339 18200046

[34] Yasukawa K Oshiumi H Takeda M Ishihara N Yanagi Y Seya T. Mitofusin 2 inhibits mitochondrial antiviral signaling. Sci Signaling (2009) 2. doi: 10.1126/scisignal.2000287 19690333

[35] Koshiba T Yasukawa K Yanagi Y Kawabata S-I. Mitochondrial membrane potential is required for MAVS-mediated antiviral signaling. Sci Signaling (2011) 4. doi: 10.1126/scisignal.2001147 21285412

[36] West AP Shadel GS Ghosh S. Mitochondria in innate immune responses. Nat Rev Immunol (2011) 11:389–402. doi: 10.1038/nri2975 21597473 PMC4281487

[37] Pourcelot M Arnoult D. Mitochondrial dynamics and the innate antiviral immune response. FEBS J (2014) 281:3791–802. doi: 10.1111/febs.12940 25051991

[38] Shah PS Link N Jang GM Sharp PP Zhu T Swaney DL. Comparative flavivirus-host protein interaction mapping reveals mechanisms of dengue and Zika virus pathogenesis. Cell (2018) 175:1931–45.e1918. doi: 10.1016/j.cell.2018.11.028 30550790 PMC6474419

[39] Lee JH Oh SJ Yun J Shin OS. Nonstructural protein NS1 of influenza virus disrupts mitochondrial dynamics and enhances mitophagy via ULK1 and BNIP3. Viruses (2021) 13. doi: 10.3390/v13091845 PMC8473137 34578425

[40] Oh SJ Lim BK Yun J Shin OS. CVB3-mediated Mitophagy plays an important role in viral replication via abrogation of interferon pathways. Front Cell Infect Microbiol (2021) 11:704494. doi: 10.3389/fcimb.2021.704494 34295842 PMC8292102

[41] Agarwal A Alam MF Basu B Pattanayak S Asthana S Syed GH. Japanese encephalitis virus NS4A protein interacts with PTEN-induced kinase 1 (PINK1) and promotes mitophagy in infected cells. Microbiol Spectr (2022) 10:e00830–00822. doi: 10.1128/spectrum.00830-22 PMC9241661 35604158

[42] Chatel-Chaix L Cortese M Romero-Brey I Bender S Neufeldt CJ Fischl W. Dengue virus perturbs mitochondrial morphodynamics to dampen innate immune responses. Cell Host Microbe (2016) 20:342–56. doi: 10.1016/j.chom.2016.07.008 PMC7105029 27545046

[43] Barbier V Lang D Valois S Rothman AL Medin CL. Dengue virus induces mitochondrial elongation through impairment of Drp1-triggered mitochondrial fission. Virology (2017) 500:149–60. doi: 10.1016/j.virol.2016.10.022 PMC5131733 27816895

[44] Muñoz-Jordán JL Sánchez-Burgos GG Laurent-Rolle M García-Sastre A. Inhibition of interferon signaling by dengue virus. Proc Natl Acad Sci (2003) 100:14333–8. doi: 10.1073/pnas.2335168100 PMC283592 14612562

[45] Best SM Morris KL Shannon JG Robertson SJ Mitzel DN Park GS. Inhibition of interferon-stimulated JAK-STAT signaling by a tick-borne flavivirus and identification of NS5 as an interferon antagonist. J Virol (2005) 79:12828–39. doi: 10.1128/JVI.79.20.12828-12839.2005 PMC1235813 16188985

[46] Lin R-J Chang B-L Yu H-P Liao C-L Lin Y-L. Blocking of interferon-induced Jak-Stat signaling by Japanese encephalitis virus NS5 through a protein tyrosine phosphatase-mediated mechanism. J Virol (2006) 80:5908–18. doi: 10.1128/JVI.02714-05 PMC1472572 16731929

[47] Ding Q Cao X Lu J Huang B Liu Y-J Kato N. Hepatitis C virus NS4B blocks the interaction of STING and TBK1 to evade host innate immunity. J Hepatol (2013) 59:52–8. doi: 10.1016/j.jhep.2013.03.019 23542348

[48] Yi G Wen Y Shu C Han Q Konan KV Li P. Hepatitis C virus NS4B can suppress STING accumulation to evade innate immune responses. J Virol (2016) 90:254–65. doi: 10.1128/JVI.01720-15 PMC4702547 26468527

[49] Kim SJ Khan M Quan J Till A Subramani S Siddiqui A. Hepatitis B virus disrupts mitochondrial dynamics: induces fission and mitophagy to attenuate apoptosis. PLoS Pathog (2013) 9:e1003722. doi: 10.1371/journal.ppat.1003722 24339771 PMC3855539

[50] Jassey A Liu C-H Changou CA Richardson CD Hsu H-Y Lin L-T. Hepatitis C virus non-structural protein 5A (NS5A) disrupts mitochondrial dynamics and induces mitophagy. Cells (2019) 8:290. doi: 10.3390/cells8040290 30934919 PMC6523690

[51] Mclean JE Wudzinska A Datan E Quaglino D Zakeri Z. Flavivirus NS4A-induced autophagy protects cells against death and enhances virus replication. J Biol Chem (2011) 286:22147–59. doi: 10.1074/jbc.M110.192500 PMC3121359 21511946

[52] Teo CSH Chu JJH. Cellular vimentin regulates construction of dengue virus replication complexes through interaction with NS4A protein. J Virol (2014) 88:1897–913. doi: 10.1128/JVI.01249-13 PMC3911532 24284321

[53] Ponia SS Robertson SJ Mcnally KL Subramanian G Sturdevant GL Lewis M. Mitophagy antagonism by ZIKV reveals Ajuba as a regulator of PINK1 signaling, PKR-dependent inflammation, and viral invasion of tissues. Cell Rep (2021) 37:109888. doi: 10.1016/j.celrep.2021.109888 34706234

[54] Li J Donath S Li Y Qin D Prabhakar BS Li P. miR-30 regulates mitochondrial fission through targeting p53 and the dynamin-related protein-1 pathway. PLoS Genet (2010) 6:e1000795. doi: 10.1371/journal.pgen.1000795 20062521 PMC2793031

[55] Liu X-Y Wei B Shi H-X Shan Y-F Wang C. Tom70 mediates activation of interferon regulatory factor 3 on mitochondria. Cell Res (2010) 20:994–1011. doi: 10.1038/cr.2010.103 20628368

[56] Dong X Yang Y Zou Z Zhao Y Ci B Zhong L. Sorting nexin 5 mediates virus-induced autophagy and immunity. Nature (2021) 589:456–61. doi: 10.1038/s41586-020-03056-z PMC7856200 33328639

[57] Lemasters JJ. Variants of mitochondrial autophagy: Types 1 and 2 mitophagy and micromitophagy (Type 3). Redox Biol (2014) 2:749–54. doi: 10.1016/j.redox.2014.06.004 PMC4085350 25009776

[58] Zou X Yuan M Zhang T Zheng N Wu Z. EVs containing host restriction factor IFITM3 inhibited ZIKV infection of fetuses in pregnant mice through trans-placenta delivery. Mol Therapy-Nucleic Acids (2021) 29:176–90. doi: 10.1016/j.ymthe.2020.09.026 PMC7791082 33002418

[59] Zhang R Fu Y Cheng M Ma W Zheng N Wang Y. sEVsRVG selectively delivers antiviral siRNA to fetus brain, inhibits ZIKV infection and mitigates ZIKV-induced microcephaly in mouse model. Mol Therapy-Nucleic Acids (2022) 30:2078–91. doi: 10.1016/j.ymthe.2021.10.009 PMC9092305 34762817

