# Supplementary material for: Zika virus modulates mitochondrial dynamics, mitophagy, and mitochondria-derived vesicles to facilitate viral replication in trophoblast cells
Source: Front Immunol. 2023 Sep 14;14:1203645. doi: 10.3389/fimmu.2023.1203645 (PMC10539660; doi:10.3389/fimmu.2023.1203645)
Supplement: Supplementary file 1 [file DataSheet_1.docx]

Supplementary Material

Zika virus modulates mitochondrial dynamics, mitophagy, and mitochondria-derived vesicles to facilitate viral replication in trophoblast cells

**Jae Kyung Lee^1^ and Ok Sarah Shin^1^***

^1^BK21 Graduate Program, Department of Biomedical Sciences, College of Medicine, Korea University Guro hospital, Seoul, Republic of Korea

*** Correspondence:**oshin@korea.ac.kr (O.S.S.); Tel.: 82-2-2626-3280

**Supplementary Figures**

**Supplementary Figure 1. ZIKV induces mitochondrial fission in JEG-3.**

JEG-3 cells were infected with mock (m) or PRVABC59 (PRV) (MOI 10) for 24 h. MitoTracker Green (500 nM) staining of mock- vs. PRV-infected JEG-3 cells following modulation of mitochondrial dynamics via Drp1- or MFN2-specific siRNA transfection or mdivi-1 (10 μM, 2h) treatment. Sites of PRV-induced mitochondrial fission are indicated with *. Scale bar = 10 μm.


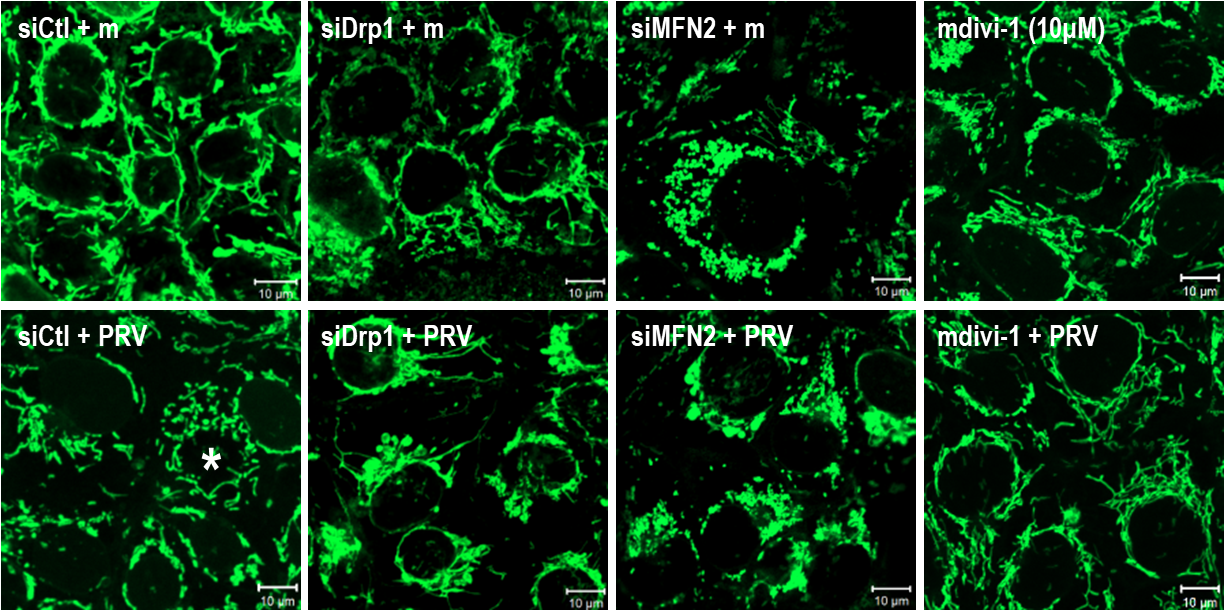


**Supplementary Figure 2. ZIKV NS4A interacts with LC3 to induce autophagy & facilitate ZIKV replication in JEG-3.** (A) JEG-3 cells expressing LC3-GFP were infected with mock (m) or PRVABC59 (PRV) (MOI 10) and dsRNA was visualized using immunofluorescence microscopy. CCCP treatment (25 μM, 2h) was included as a control for LC3 puncta formation in mitophagy-induced cells. Scale bar = 10 μm. (B, C) qRT-PCR analysis was carried out to determine ZIKV vRNA levels in JEG-3 cells. Rapamycin (B) or 3-MA (C) was treated for 2 h prior to infection with PRVABC59 to enhance or suppress autophagic activity. (D) Western blot analysis of autophagy markers like LC3 and p62 in ZIKV NS4A- or NS4B-transfected JEG-3 cells. LC3 II:I ratio was quantified using ImageJ software. (E) Confocal microscopy of JEG-3 cells co-transfected with LC3 and ZIKV NS4A or NS4B. CCCP (25 μM, 2h) treatment was included as a positive control for autophagy induction. Scale bar = 10 μm. Number of LC3 puncta per cell were calculated using 10 random fields (graph). Data represented as mean + SD. *p<0.05, **p<0.01, and ***p<0.001, when compared to the control. (F) JEG-3 cells transfected with LC3-GFP and FLAG-tagged ZIKV NS4A or NS4B were lysed and precipitated using anti-FLAG antibody. Western blot analysis of cell lysates and immunoprecipitates using anti-FLAG and anti-GFP antibodies. Whole cell lysates (WCL)


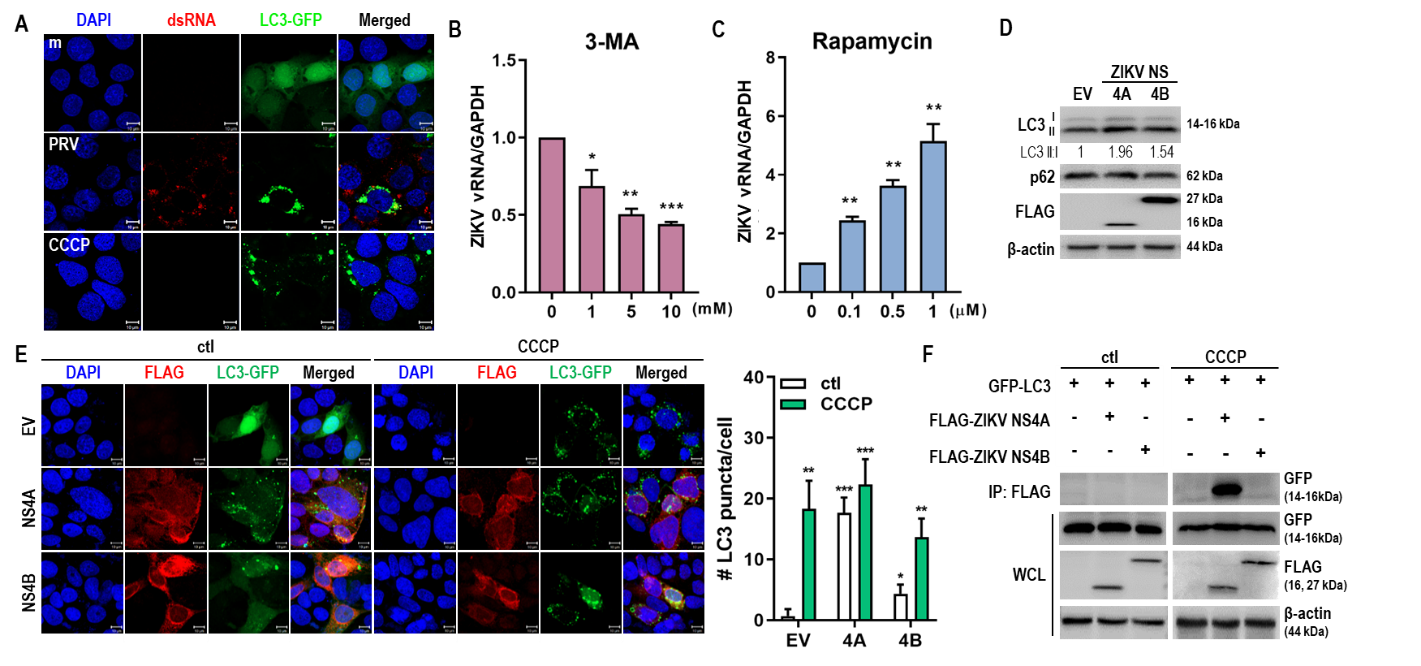


**Supplementary Figure 3. ZIKV NS4A induces mitophagy in JEG-3 cells.** JEG-3 cells were co-transfected with mtKeima- and empty vector (EV)- or ZIKV NS4A-encoding plasmids, and treated with CCCP (25 μM, 2h) prior to imaging. Fluorescence signals obtained following excitation with 488 nm (green) and 561 nm (red) are shown. Scale bar = 10 μm.


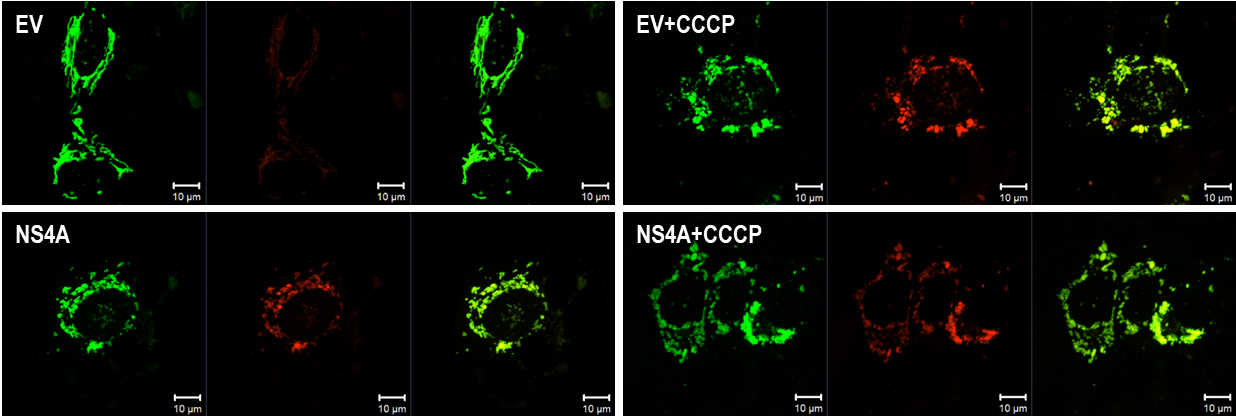


**Supplementary Figure 4. ZIKV NS4A does not affect apoptotic signaling pathway.** JEG-3 cells were treated with DMSO vs. staurosporine (STS; 5 μM, 6h) following transfection with ZIKV NS4A or NS4B. Western blot analysis of apoptosis-associated PARP and caspase 3 expression and cleavage levels (A). Cytotoxicity levels were measured and compared between DMSO- and STS-treated samples (B). Data represented as mean + SD. **p<0.01, and ***p<0.001, when compared to the DMSO control.


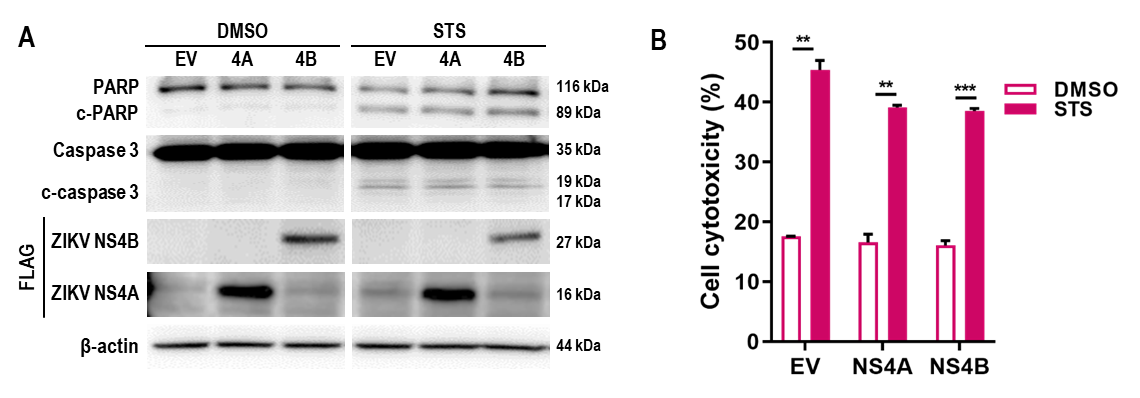


**Supplementary Figure 5. Proteomics profiling of ZIKV-infected sEVs.** sEV protein was isolated from mock- vs. PRVABC59-infected JEG-3 cells and subject to liquid chromatography/mass spectrometry (LC/MS-MS)-based sEV proteomics profiling. (A) Heatmap of sEV proteins detected in mock vs. PRVABC59-infected cells. (B) Gene annotation enrichment analysis of protein-encoding genes was performed according to the subcellular localization of sEV proteins. (C) Heatmap of sEV proteins that can localize to the mitochondria. (D) Heatmap of sEV proteins that can be secreted.


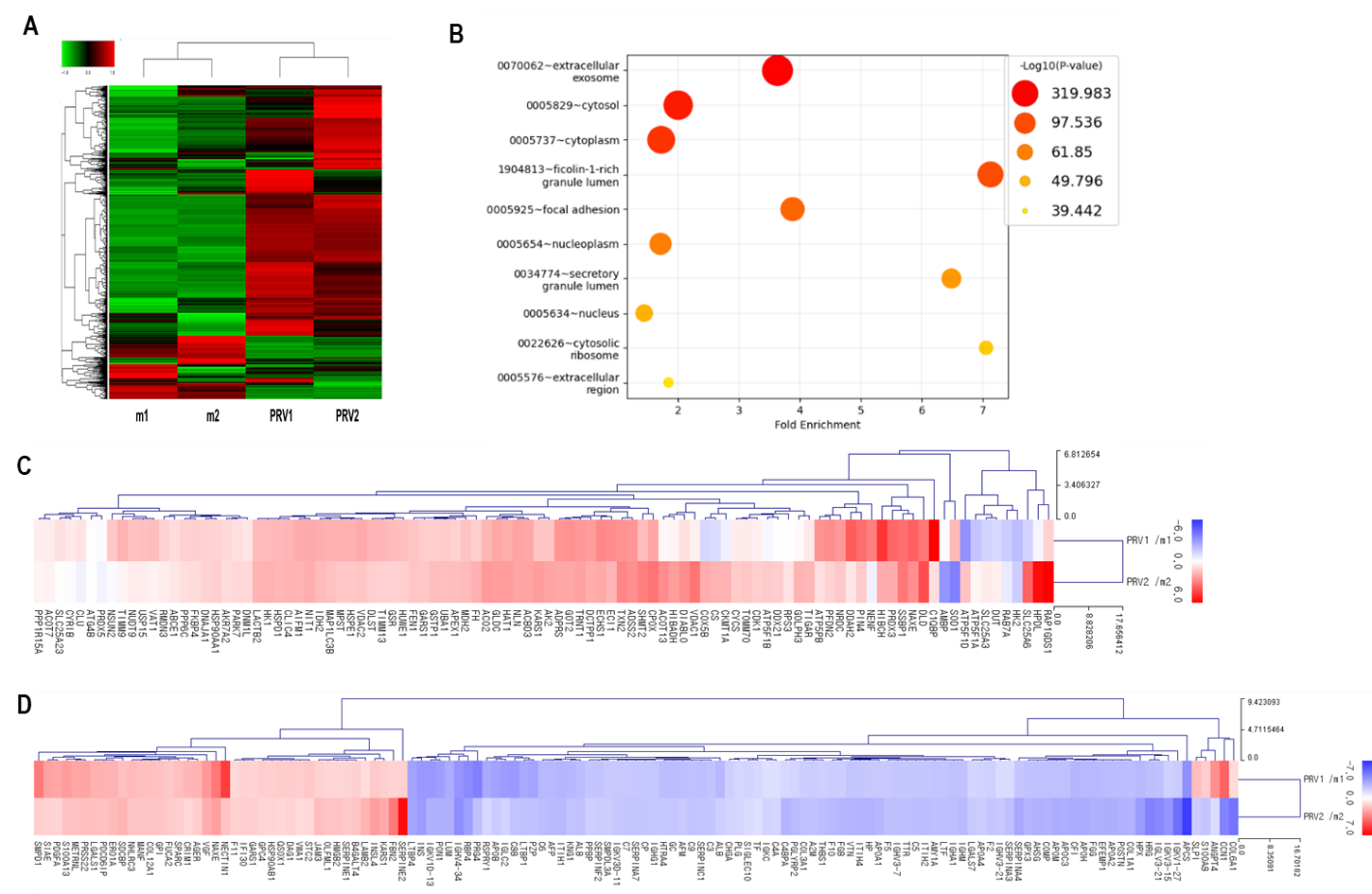


**Supplementary Figure 6. Small RNA sequencing reveal miRNA profiles of ZIKV-infected sEVs.** sEVs were isolated from mock- vs. PRVABC59-infected JEG-3 cells. Total RNA was extracted from sEVs and subject to small RNA sequencing for secretory miRNA profiling. (A) PCA plot of sEVs based on their similarity in terms of miRNA composition. (B) Heatmap representation of sEV miRNAs whose expression are regulated during ZIKV infection of JEG-3 cells.

**
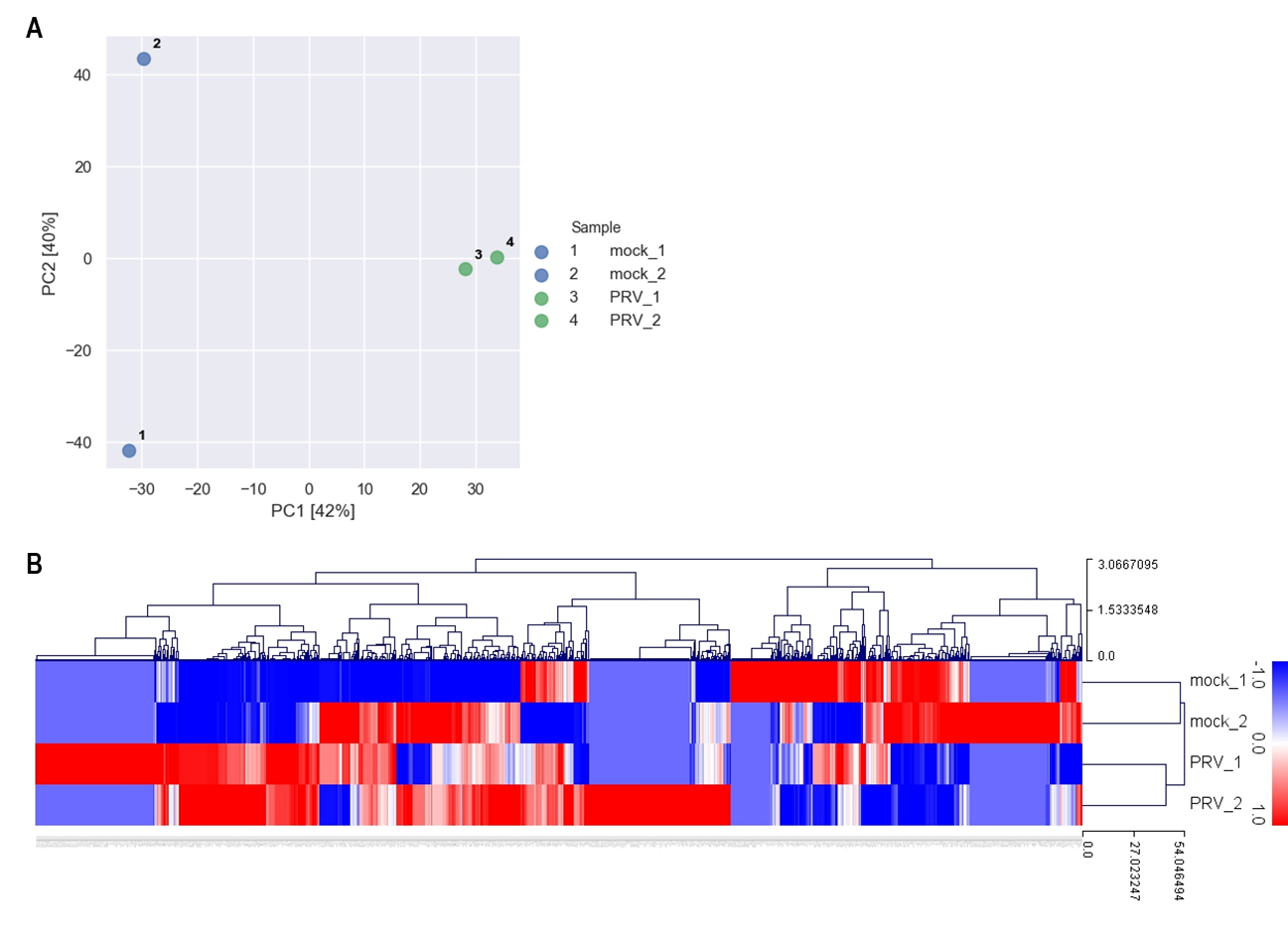
**

**Supplementary Tables**

**Supplementary Table 1. List of primers used in the study**

| **Gene symbol** | **Forward** | **Reverse** |
| --- | --- | --- |
| 1. ***ZIKV vRNA*** | AGATGACTGCGTTGTGAAGC | GAGCAGAACGGGACTTCTTC |
| 1. ***Drp1*** | CACTTGTGGATTTGCCAGGAATGACC | TGCGACCATCTGGATCTACCTCTCTT |
| 1. ***FIS1*** | CATCGTGCTGCTGGAGAGC | GCAGAGAGCAGGTGAGGCTG |
| 1. ***BNIP3*** | CAGGGCTCCTGGGTAGAACT | CTACTCCGTCCAGACTCATGC |
| 1. ***NIX*** | TTGGATGCACAACATGAATCAGG | TCTTCTGACTGAGAGCTATGGTC |
| 1. ***IFN-β*** | GCTTGGATTCCTACAAAGAAGCA | ATAGATGGTCAATGCGGCGTC |
| 1. ***ISG15*** | GAGAGGCAGCGAACTCATCT | CTTCAGCTCTGACACCGACA |
| 1. ***OAS1*** | TGTCCAAGGTGGTAAAGGGTG | CCGGCGATTTAACTGATCCTG |
| 1. ***mtDNA*** | GATTTGGGTACCACCCAAGTATTG | GTACAATATTCATGGTGGCTGGCA |
| 1. ***18s rRNA*** | TAGAGGGACAAGTGGCGTTC | CGCTGAGCCAGTCAGTGT |
| 1. ***GAPDH*** | TGAACGGGAAGCTCACTGG | TCCACCACCCTGTTGCTGTA |
