# Supplementary material for: Zika virus modulates mitochondrial dynamics, mitophagy, and mitochondria-derived vesicles to facilitate viral replication in trophoblast cells
Source: Front Immunol. 2023 Sep 14;14:1203645. doi: 10.3389/fimmu.2023.1203645 (PMC10539660; doi:10.3389/fimmu.2023.1203645)
Supplement: Supplementary file 2 [file Presentation_1.pptx]

## Slide 1
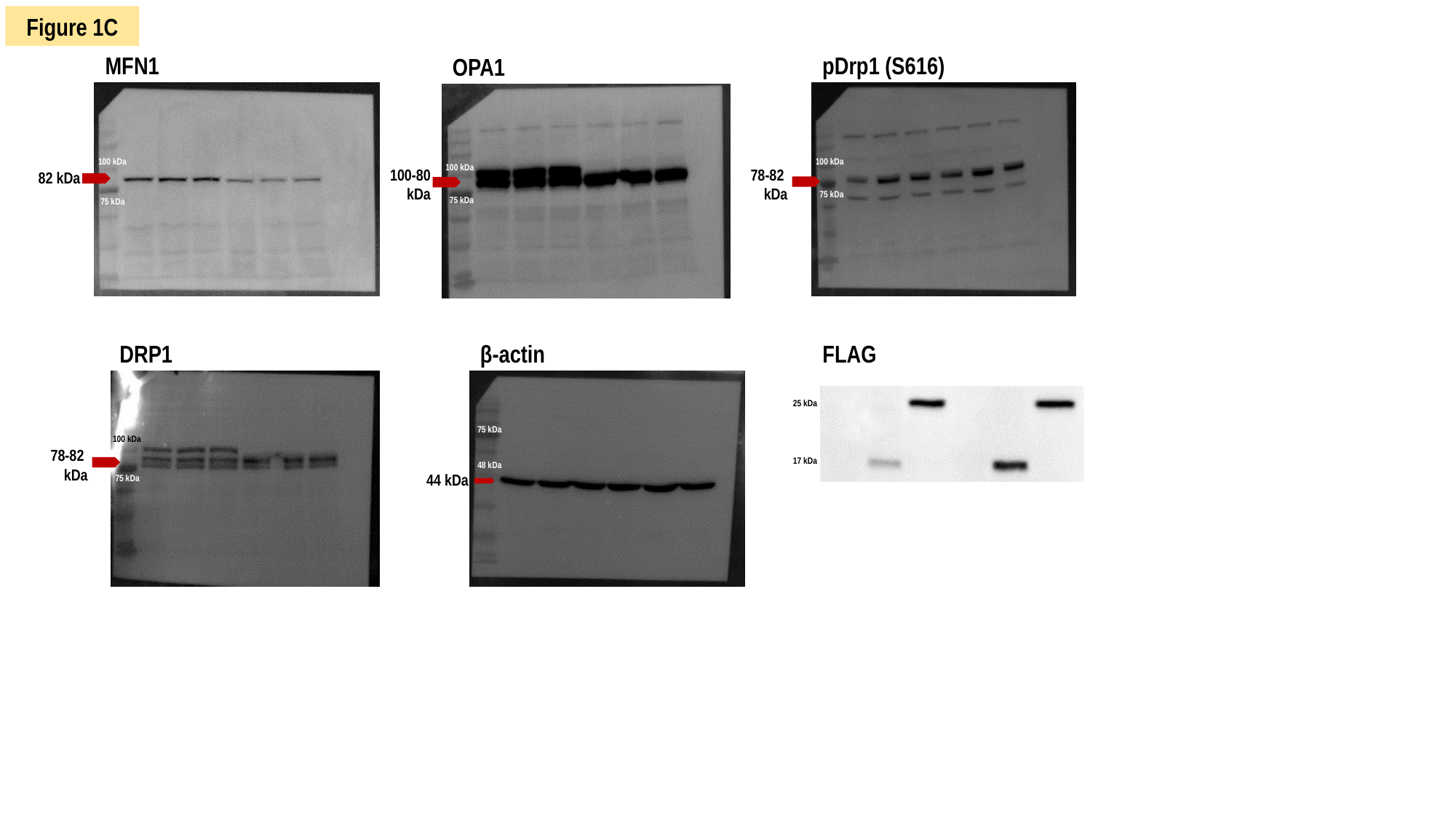

Figure 1C
pDrp1 (S616)
MFN1
OPA1
100 kDa
100 kDa
100 kDa
78-82
kDa
100-80 kDa
82 kDa
75 kDa
75 kDa
75 kDa
DRP1
β-actin
FLAG
25 kDa
75 kDa
100 kDa
78-82
kDa
17 kDa
48 kDa
44 kDa
75 kDa

## Slide 2
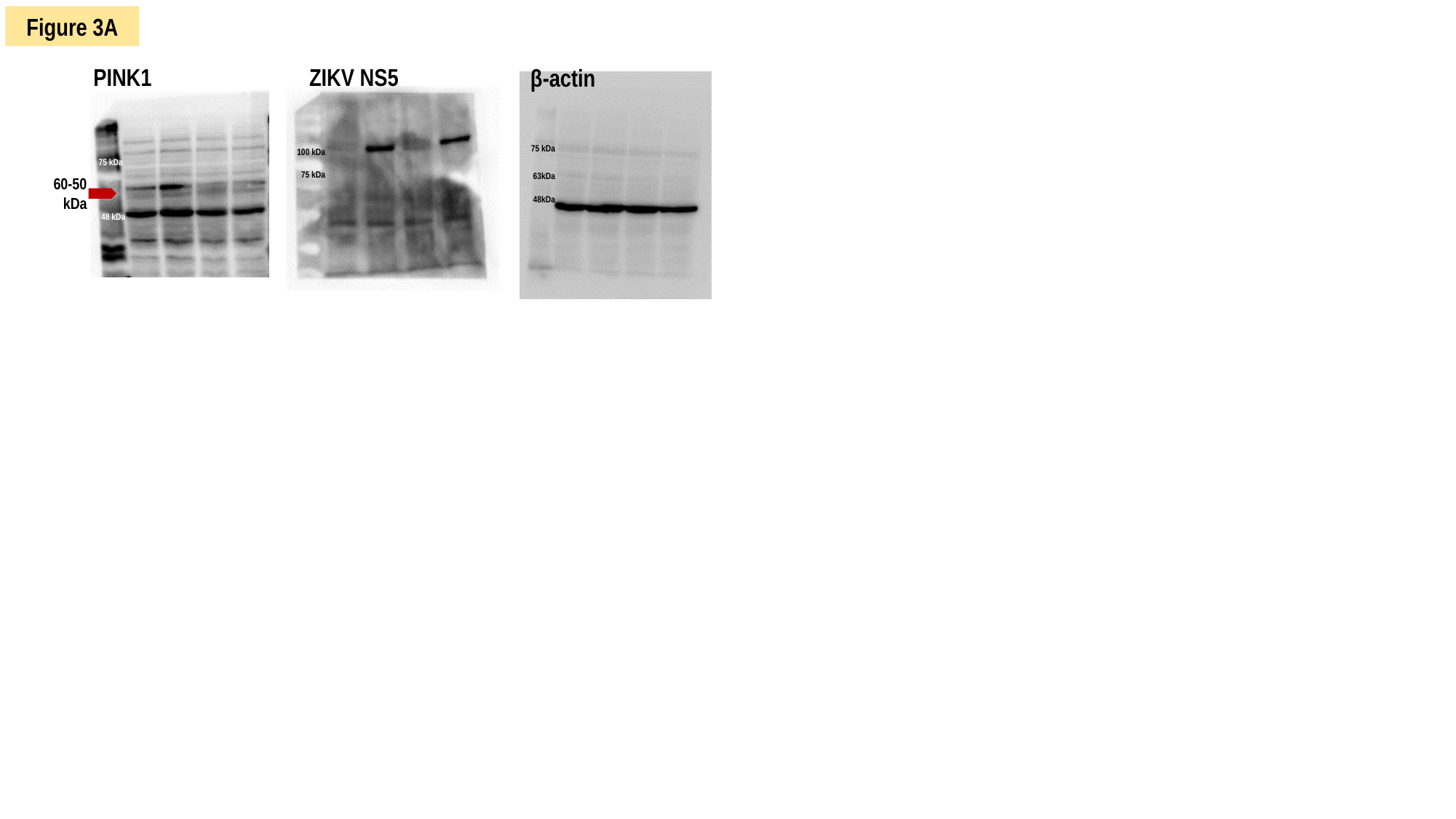

Figure 3A
PINK1
ZIKV NS5
β-actin
75 kDa
100 kDa
75 kDa
75 kDa
63kDa
60-50 kDa
48kDa
48 kDa

## Slide 3
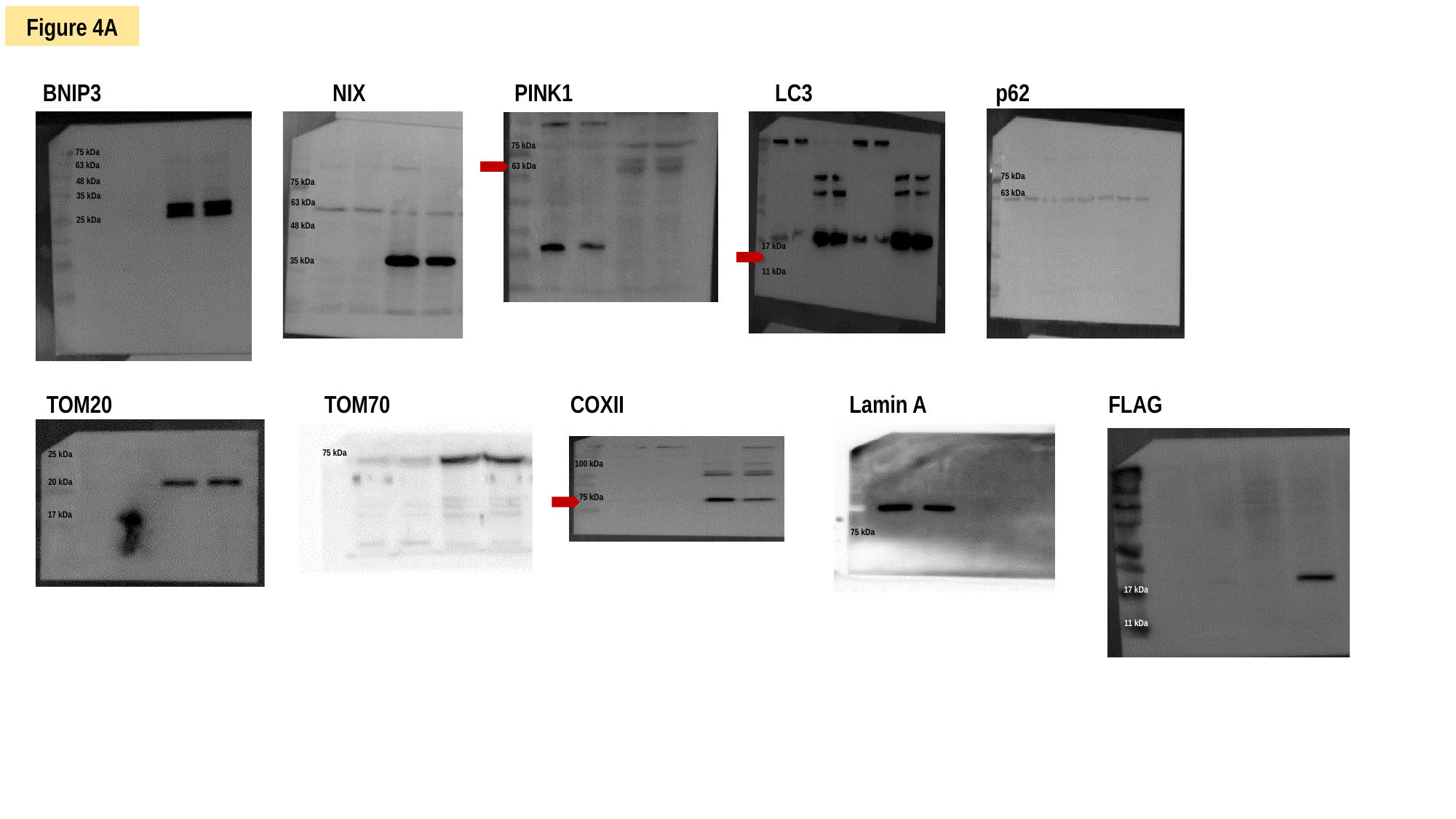

Figure 4A
BNIP3
p62
NIX
LC3
PINK1
75 kDa
75 kDa
63 kDa
63 kDa
75 kDa
48 kDa
75 kDa
63 kDa
35 kDa
63 kDa
25 kDa
48 kDa
17 kDa
35 kDa
11 kDa
TOM20
TOM70
COXII
Lamin A
FLAG
75 kDa
25 kDa
100 kDa
20 kDa
75 kDa
17 kDa
75 kDa
17 kDa
11 kDa

## Slide 4
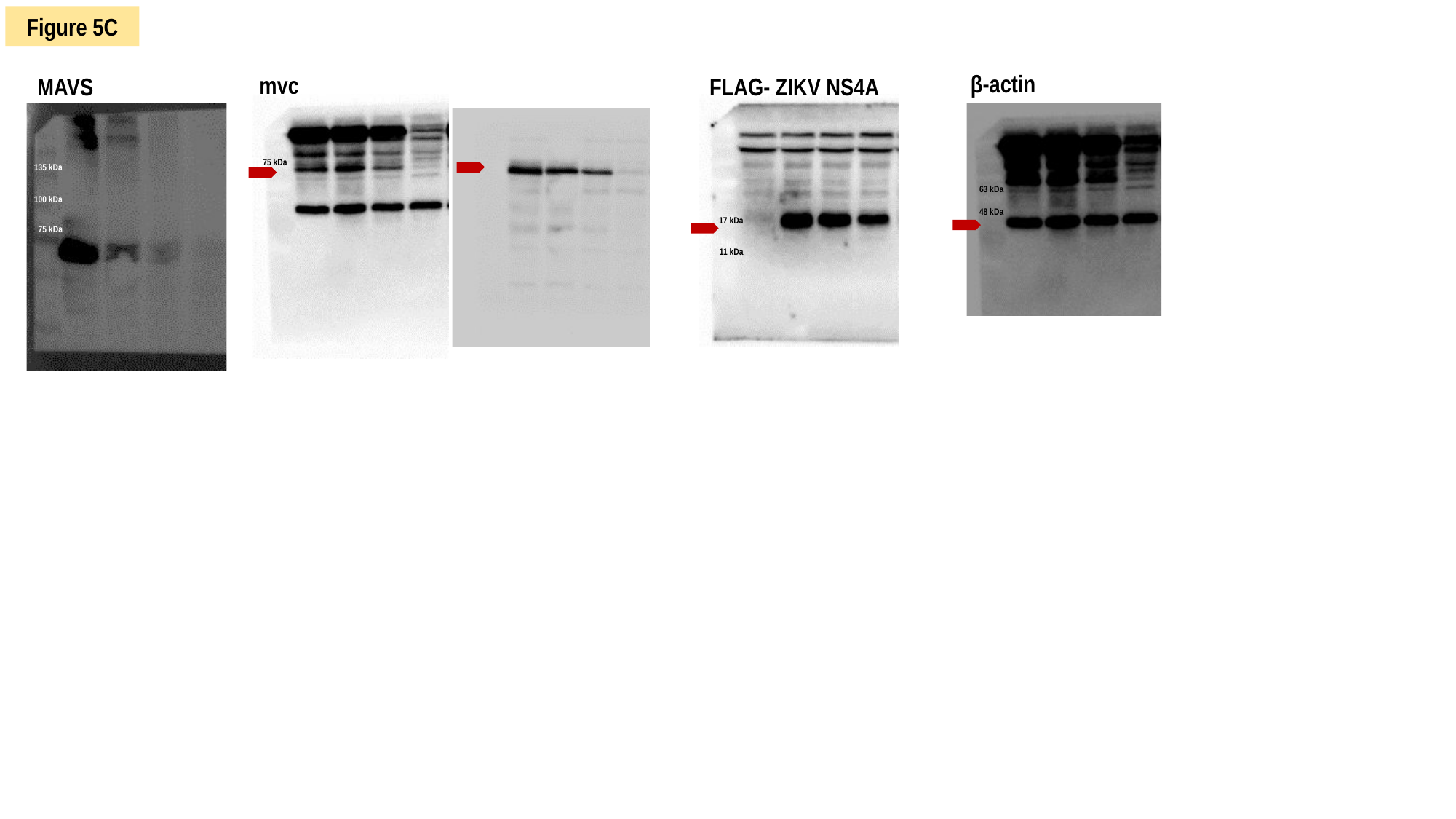

Figure 5C
β-actin
myc
MAVS
FLAG- ZIKV NS4A
75 kDa
135 kDa
63 kDa
100 kDa
48 kDa
17 kDa
75 kDa
11 kDa

## Slide 5
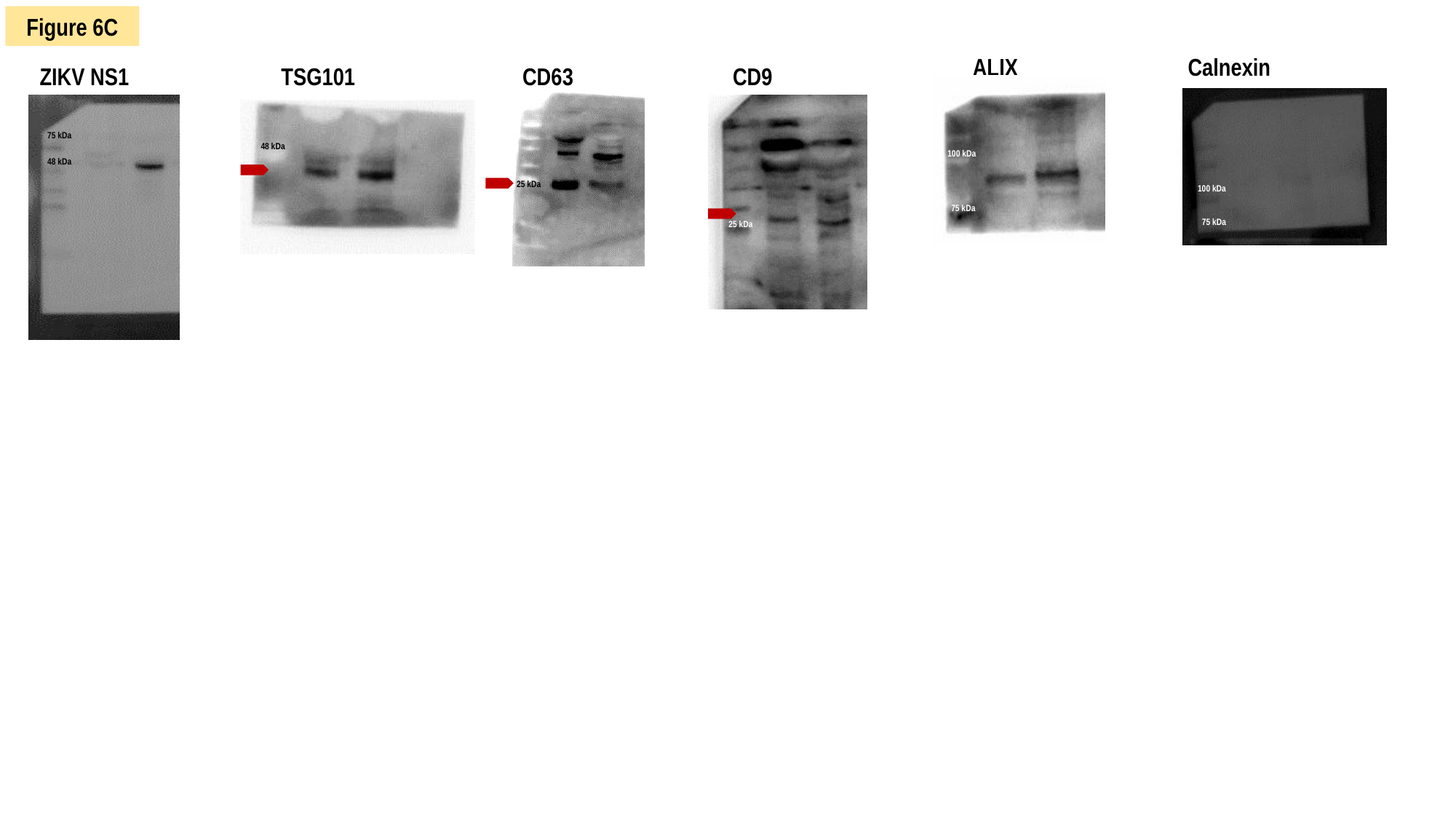

Figure 6C
ALIX
Calnexin
ZIKV NS1
TSG101
CD63
CD9
75 kDa
48 kDa
100 kDa
48 kDa
25 kDa
100 kDa
75 kDa
75 kDa
25 kDa

## Slide 6
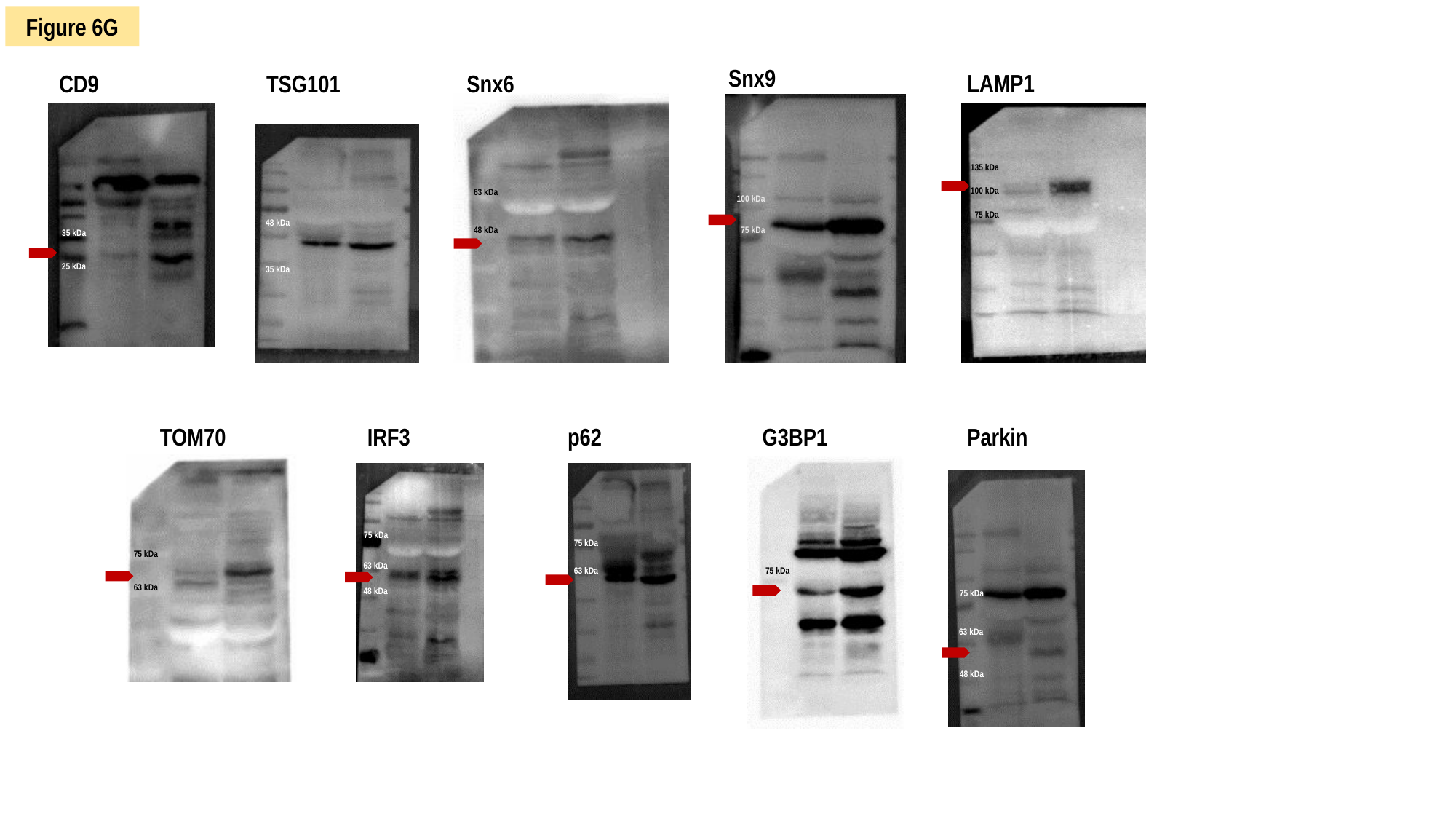

Figure 6G
Snx9
LAMP1
CD9
TSG101
Snx6
135 kDa
100 kDa
63 kDa
100 kDa
75 kDa
48 kDa
48 kDa
75 kDa
35 kDa
25 kDa
35 kDa
TOM70
IRF3
p62
G3BP1
Parkin
75 kDa
75 kDa
75 kDa
63 kDa
63 kDa
75 kDa
63 kDa
48 kDa
75 kDa
63 kDa
48 kDa

## Slide 7
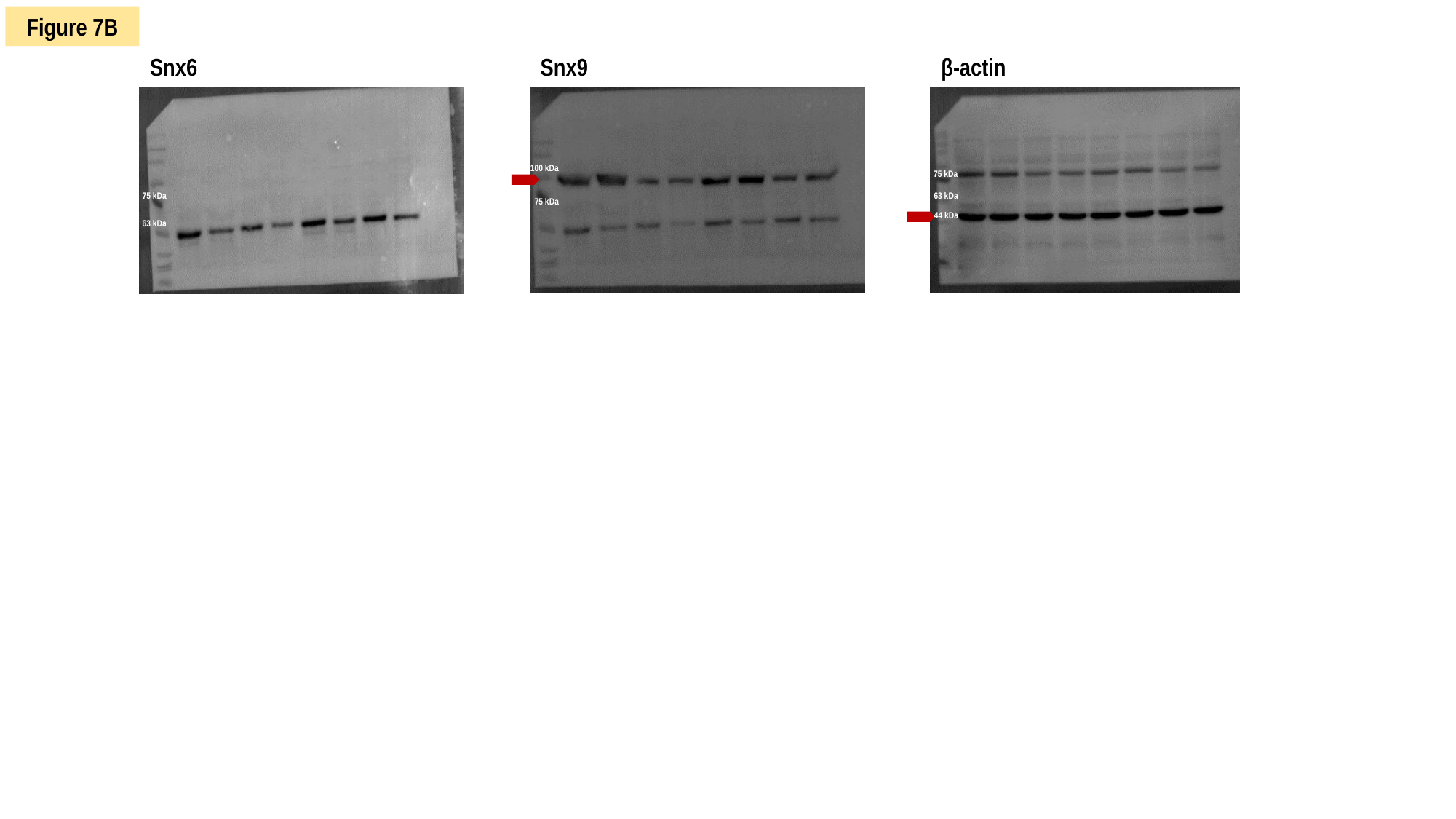

Figure 7B
Snx9
β-actin
Snx6
100 kDa
75 kDa
63 kDa
75 kDa
75 kDa
44 kDa
63 kDa

## Slide 8
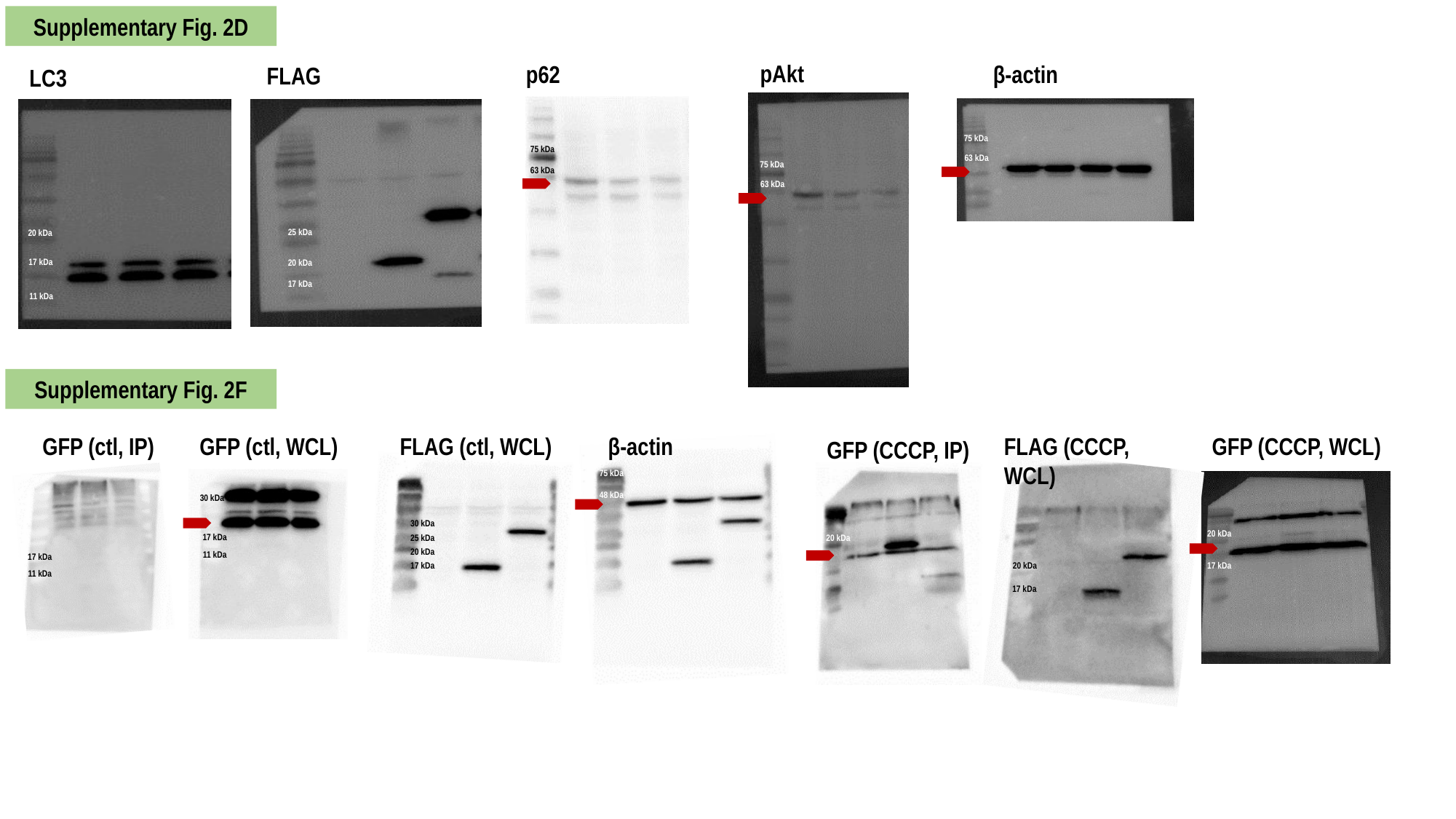

Supplementary Fig. 2D
pAkt
p62
β-actin
FLAG
LC3
75 kDa
75 kDa
63 kDa
75 kDa
63 kDa
63 kDa
25 kDa
20 kDa
17 kDa
20 kDa
17 kDa
11 kDa
Supplementary Fig. 2F
GFP (ctl, WCL)
FLAG (ctl, WCL)
β-actin
GFP (CCCP, WCL)
FLAG (CCCP, WCL)
GFP (ctl, IP)
GFP (CCCP, IP)
75 kDa
48 kDa
30 kDa
30 kDa
20 kDa
17 kDa
20 kDa
25 kDa
20 kDa
11 kDa
17 kDa
17 kDa
17 kDa
20 kDa
11 kDa
17 kDa

## Slide 9
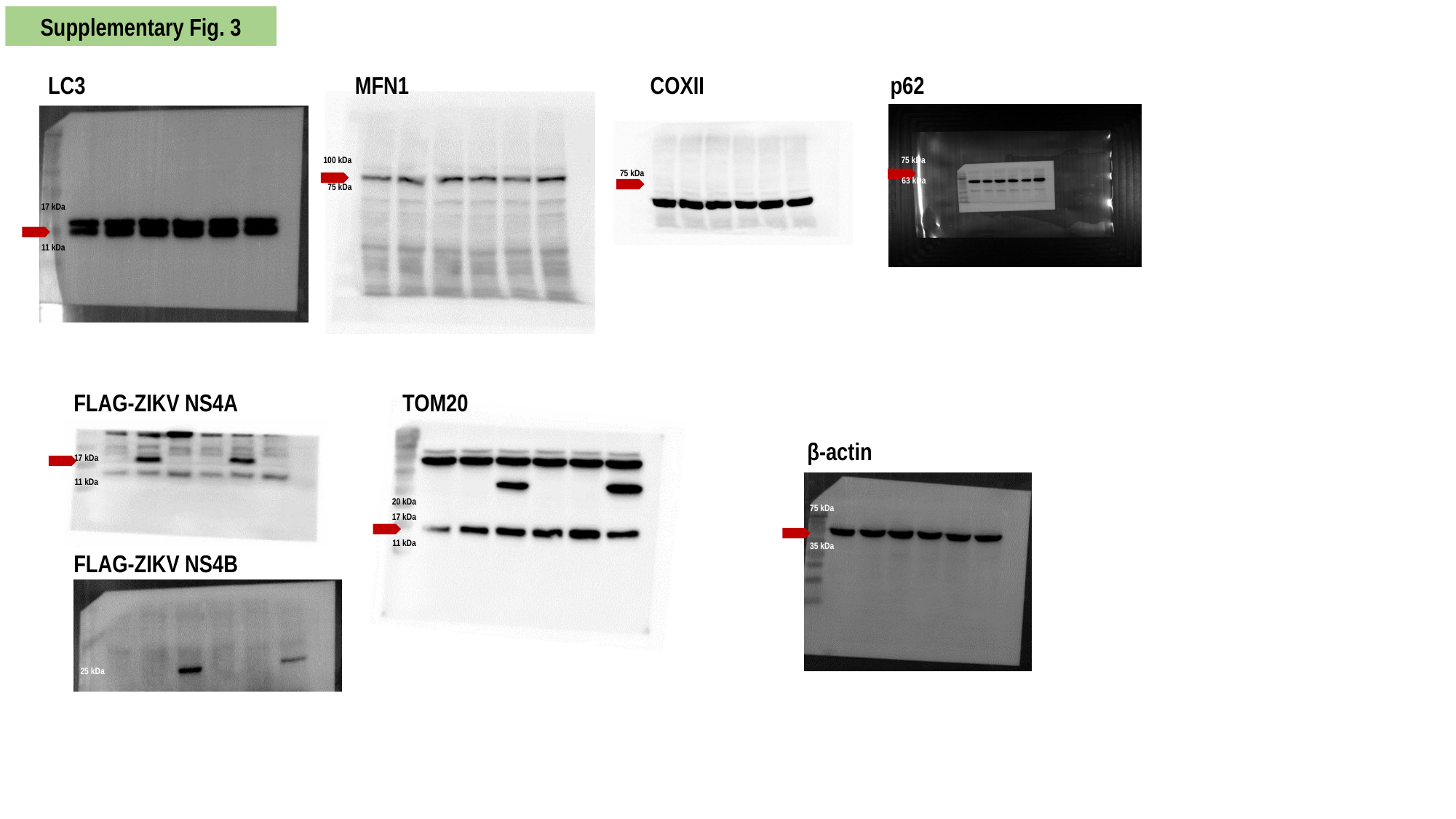

Supplementary Fig. 3
LC3
MFN1
COXII
p62
75 kDa
100 kDa
75 kDa
63 kDa
75 kDa
17 kDa
11 kDa
FLAG-ZIKV NS4A
TOM20
β-actin
17 kDa
11 kDa
20 kDa
75 kDa
17 kDa
11 kDa
35 kDa
FLAG-ZIKV NS4B
25 kDa

## Slide 10
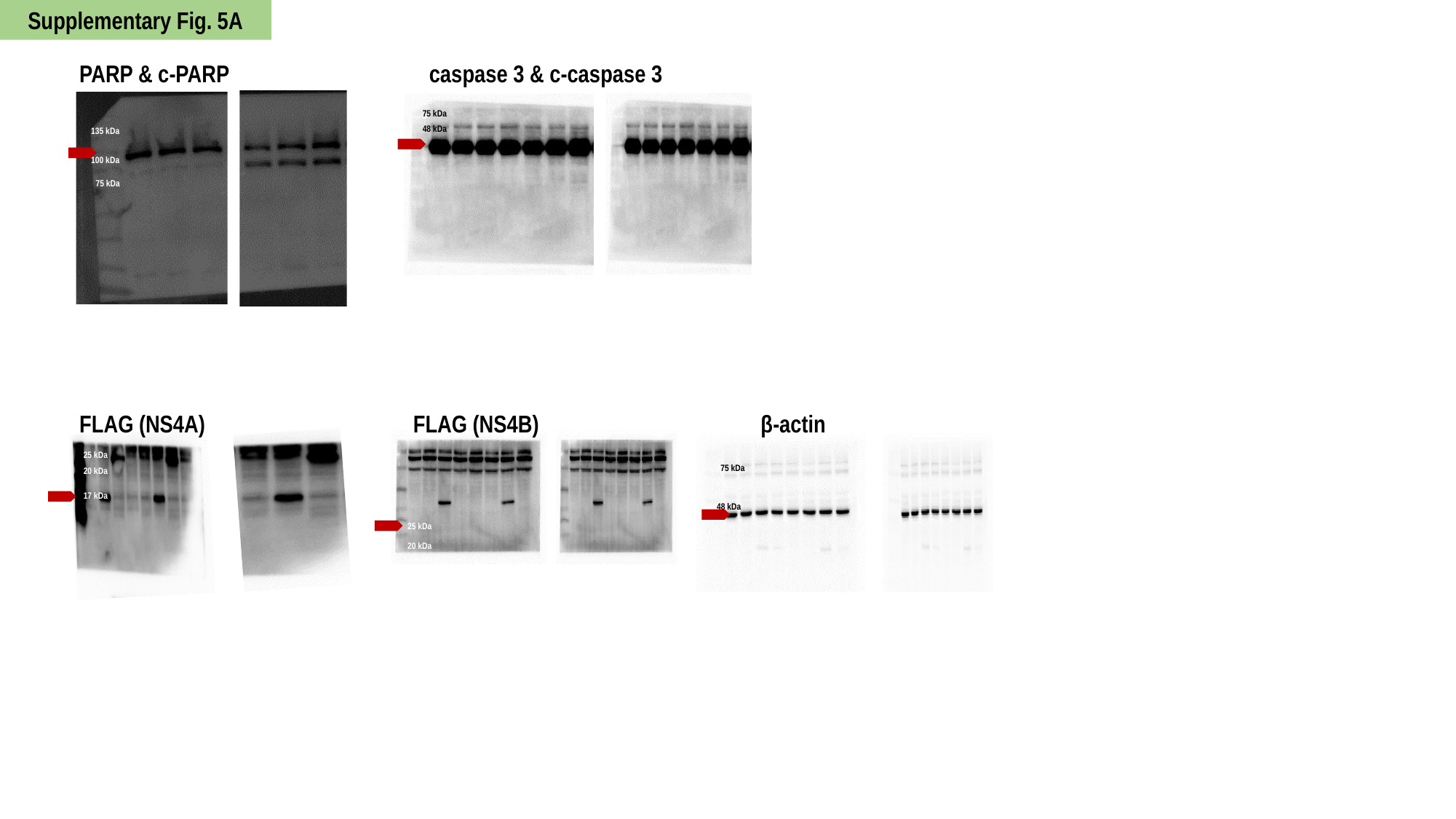

Supplementary Fig. 5A
PARP & c-PARP
caspase 3 & c-caspase 3
75 kDa
48 kDa
135 kDa
100 kDa
75 kDa
FLAG (NS4A)
FLAG (NS4B)
β-actin
25 kDa
75 kDa
20 kDa
17 kDa
48 kDa
25 kDa
20 kDa
